# Data for iTRAQ secretomic analysis of *Aspergillus fumigatus* in response to different carbon sources

**DOI:** 10.1016/j.dib.2015.03.001

**Published:** 2015-03-11

**Authors:** Sunil S. Adav, Anita Ravindran, Siu Kwan Sze

**Affiliations:** a School of Biological Sciences, Nanyang Technological University, 60 Nanyang Drive, Singapore 637551, Singapore

**Keywords:** *Aspergillus fumigates*, Bioenergy, Biorefinery, Cellulases, Deamidation

## Abstract

Here, we provide data related to the research article entitled “Quantitative proteomics study of *Aspergillus fumigatus* secretome revealed deamidation of secretory enzymes” by Adav et al. (J. Proteomics (2015) [1]). *Aspergillus* sp. plays an important role in lignocellulosic biomass recycling. To explore biomass hydrolyzing enzymes of *A. fumigatus*, we profiled secretome under different carbon sources such as glucose, cellulose, xylan and starch by high throughput quantitative proteomics using isobaric tags for relative and absolute quantification (iTRAQ). The data presented here represents the detailed comparative abundances of diverse groups of biomass hydrolyzing enzymes including cellulases, hemicellulases, lignin degrading enzymes, and peptidases and proteases; and their post translational modification like deamidation.

**Specifications table**

**Table:** 

Subject area	Biology, fungal proteomic, lignocellulosic bioenergy
More specific subject area	Secretomics of *Aspergillus fumigatus*
Type of data	Tables, MS/MS spectrums
How data was acquired	QStar Elite mass spectrometer (Applied Biosystems/MDS Sciex)
Data format	4-Plex iTRAQ reagent multiplex kit (Applied Biosystems, Foster City, CA)
	ERLIC chromatography
	QStar Elite mass spectrometer (Applied Biosystems/MDS Sciex) coupled with online microflow HPLC system
	Data acquisition was performed with Analyst QS 2.0 software (Applied Biosystems/MDS SCIEX).
	Data processing by ProteinPilot™ software 3.0 (revision number
	114732; Applied Biosystems, Foster City, CA).
	Database, concatenated JGI downloaded from (http://www.aspgd.org/, http://genome.jgi.doe.gov/Aspfu1/Aspfu1.home.html)
Experimental factors	Secreted proteins of *Aspergillus fumifatus* LF9 cultivated on glucose, cellulose, xylan and starch were collected by centrifugation and concentrated by lyophilization. Proteins were precipitated by ice-cold acetone. The proteins were digested, iTRAQ-labeled, fractionated and analyzed using QStar Elite mass spectrometer.
Experimental features	Isobaric tags for relative and absolute quantification (iTRAQ)
Data source location	Singapore
Data accessibility	Analyzed data sets are available with this article.

## Value of the data

• The data provides relative quantitative information of secretory proteins of differential response by *Aspergillus fumigatus* to different carbon source.
• The data provide insight into secretion of cellulosic biomass hydrolyzing enzymes of *Aspergillus fumigatus.*
• iTRAQ data provides information on the abundance of the biomass degrading enzymes for design and optimization of industrial enzyme cocktail.

## 1 Data, experimental design, materials and methods

Fungal secretome were extracted and processed according to our earlier protocol [2]. The cultivation of *A. fumigatus* LF9 under different carbon sources such as glucose, cellulose, xylan; this study quantified diverse group of hemicellulases including endo-1,4-beta-xylanase, beta-xylosidase, alpha-1,2-mannosidase, alpha-l-arabinofuranosidase, arabinanase, beta-galactosidase, acetyl xylan esterase, rhamnogalacturonan acetylesterase, and many more as listed in Supplementary Table S1. Enzymes like laccase, isoamyl alcohol oxidase, glutathione reductase, oxidoreductases etc. involved in lignin degradation were also identified and quantified. Their comparative abundances under different carbon sources were listed in Supplementary Table S2. In addition to cellulases, hemicellulases and lignin degrading enzymes, this study identified several peptidases and proteases in the secretome (Supplementary Table S2). The proteins with unknown function (hypothetical proteins) were also expressed and their comparative abundances were listed in Supplementary Table S3. The cellulolytic activity of the isolated strain was further confirmed by using zymographic analysis. The band showing cellulolytic activity was cut, digested and analyzed by LC–MS/MS. The proteins identified in the band are listed in Supplementary Table S4. The data analysis revealed deamidation of key cellulose hydrolyzing enzymes, hemicellulases and several other proteins. The spectrums showing peptide sequences, fragmentation pattern and sites of modification are provided as a supplementary material (spectrum deamidation).

## 2 Microorganism cultivation and secretome extraction

The fungal isolates belonging to *Aspergillus* sp. isolated, identified and labeled as *A. fumigatus* LF9 was used in this study [3]. DNA sequence of this isolate is available in GenBank under accession no. JF815073. *A. fumigatus* LF9 was cultivated at 50 °C at 100 rpm in medium composed of ammonium sulfate 3.1 g L^−1^, sodium chloride 1.5 g L^−1^, dipotassium phosphate 1.2 g L^−1^, monopotassium phosphate 0.9 g L^−1^, magnesium sulfate 0.3 g L^−1^ and 5.0 g L^−1^ different carbon sources such as glucose, cellulose, xylan and starch. Mycelium was harvested in mid exponential phase (4 days) and supernatant was collected by centrifugation at 7500×*g* at 4 °C (Beckman Coulter, Brea, CA, USA) for 7 min. The experimental design included three flaks for each carbon source used. The supernatant henceforth called secretome was further filtered through 0.25 μm filters. Proteins were precipitated using ice cold acetone for overnight and protein content was estimated by Bradford method [4]. The strains belonging to species *A. fumigatus* are pathogenic and hence biosafety guidelines were strictly followed while working with this fungal isolate.

## 3 Protein separation, protein digestion, and peptide extraction

Proteins from each test condition were separated on 10% SDS-PAGE at 100 V, and protein bands were visualized by staining with Coomassie Brilliant blue G-250. For proteomic analysis, proteins from all four conditions were digested separately by using our optimized protocol [2,5,6]. Concisely, 200 µg proteins from each condition were loaded on SDS-PAGE and run at 100 V for 30–40 min and concentrated in separating gel. Each sample lane was sliced separately, cut into small pieces (approximately 1 mm^2^) washed with 75% acetonitrile (ACN) containing triethylammonium bicarbonate buffer (TEAB, 25 mM) and then distained using TEAB (25 mM) alone and TEAB with 50% ACN. Then gel pieces were reduced with Tris 2-carboxyethyl phosphine hydrochloride (5 mM) and alkylated with methyl methanethiosulfonate (10 mM). The gel pieces were washed twice with TEAB to remove excess reducing and alkylating agent and dehydrated using 100% ACN. The gel pieces were subjected to sequencing grade modified trypsin (Promega, Madison, WI) digestion at 37 °C for overnight. The peptides were extracted using 50% ACN plus 5% acetic acid. The extracted peptides were concentrated using concentrator (Eppendrof AG, Hamburg, Germany) for iTRAQ labeling.

## 4 iTRAQ labeling and LC–MS/MS analysis

iTRAQ labeling of peptides was performed using 4-plex iTRAQ reagent multiplex kit (Applied Biosystems, Foster City, CA) following manufacturer׳s protocol. The labeling was 113: *A. fumigatus* LF9 glucose (control); 114: *A. fumigatus* LF9 cellulose; 115: *A. fumigatus* LF9 xylan; 116: *A. fumigatus* LF9 starch. Thus, peptides from each test condition were labeled with respective isobaric tags, incubated for 2 h at room temperature (20±2 °C) and reaction was stopped by adding 100 µL water. The labeled peptides were combined together and vacuum-centrifuged to dryness. The labeled samples were acidified with 0.1% trifluoroacetic acid and de-salted using Sep-Pak C18 cartridges and then HPLC fractionated. The iTRAQ labeled peptides were dissolved in buffer A (10 mM ammonium acetate, 85% acetonitrile, 0.1% formic acid) and fractionated using ERLIC column (200×4.6 mm, 5 μm particle size, 300 Å pore size) by HPLC system (Shimadzu, Japan) at flow rate of 1.0 mL min^−1^. The eluted 60 fractions were collected using automated fraction collector, combined to 20 fractions and vacuum dried before LC–MS/MS analysis. The labeled vacuum dried peptides were reconstituted in 3% ACN with 0.1% formic acid and analyzed by QStar Elite mass spectrometer (Applied Biosystems/MDS Sciex) coupled with online microflow HPLC system (Shimadzu). The labeled peptides were separated on a home-packed nanobored C18 column with a picofrit nanospray tip (New Objectives, Wubrun, MA) coupled to the LC–MS/MS system at a constant flow rate of 300 nL min^−1^. Analysis was carried out in positive ion mode using Analyst QS 2.0 software (Applied Biosystems) and data were acquired with a selected mass range of 300–1600 *m*/*z*. Peptides with charge +2 and above were selected for MS/MS. The three most abundantly peptides above a five-count threshold were selected for MS/MS, and dynamically excluded for 30 s with 30 mDa mass tolerance. Smart information-dependent acquisition (IDA) was activated with automatic collision energy and automatic MS/MS accumulation. The fragment intensity multiplier was set to 20 and maximum accumulation time was 2 s. Data acquisition was performed with Analyst QS 2.0 software (Applied Biosystems/MDS SCIEX).

## 5 Data analysis

The data acquired by Analyst QS 2.0 software was further processed for peak list generation, protein identification, and peptide quantification using ProteinPilot™ software 3.0 (revision number 114732; Applied Biosystems, Foster City, CA). The Paragon algorithm in the ProteinPilot™ software was used for the peptide identification. The user defined parameters were as: (i) Sample type-iTRAQ 4-plex (Peptide Labeled); (ii) Cysteine alkylation-MMTS; (iii) Digestion-trypsin; (iv) Instrument-QSTAR Elite ESI; (v) Special factors—none; (vi) Species—none; (vii) Specify processing—Quantitate; (viii) ID Focus—biological modifications, amino acid substitutions; (ix) Database—concatenated JGI downloaded from (http://www.aspgd.org/, http://genome.jgi.doe.gov/Aspfu1/Aspfu1.home.html); (x) Search effort-thorough. The precursors and fragment mass tolerances were default as adopted by the software. For iTRAQ quantitation, peptides were automatically selected by Pro Group algorithm for calculations of the reporter peak area, *p*-value etc. The final data was auto bias-corrected and a strict cut-off of unused ProteinScore ≥2, which corresponds to a confidence limit of 99% was applied. Proteins quantified with at least two peptides with 95% confidence were considered for further analysis. The data was classified according to glycosyl hydrolase (GH) family following KEGG database. These proteins were sorted using N-terminal Sec-dependant secretion signal using SignalP 3.0 (http://www.cbs.dtu.dk/services/ SignalP/)[7]

## Conflict of interest

The authors declare that they have no competing interests.

## Footnotes

Appendix A Supplementary data associated with this article can be found in the online version at doi:10.1016/j.dib.2015.03.001.
