# Supplementary material for: Data for iTRAQ secretomic analysis of Aspergillus fumigatus in response to different carbon sources
Source: Data Brief. 2015 Mar 11;3:175–9. doi: 10.1016/j.dib.2015.03.001 (PMC4510139; doi:10.1016/j.dib.2015.03.001)
Supplement: Supplementary file 1 — Supplementary Data [file mmc1.zip › Spectrum_Deamidation.docx]

1. Afu1g14560| Aspergillus fumigatus alpha-mannosidase (494 aa)

N#GWGASAVDALSTAIVMR


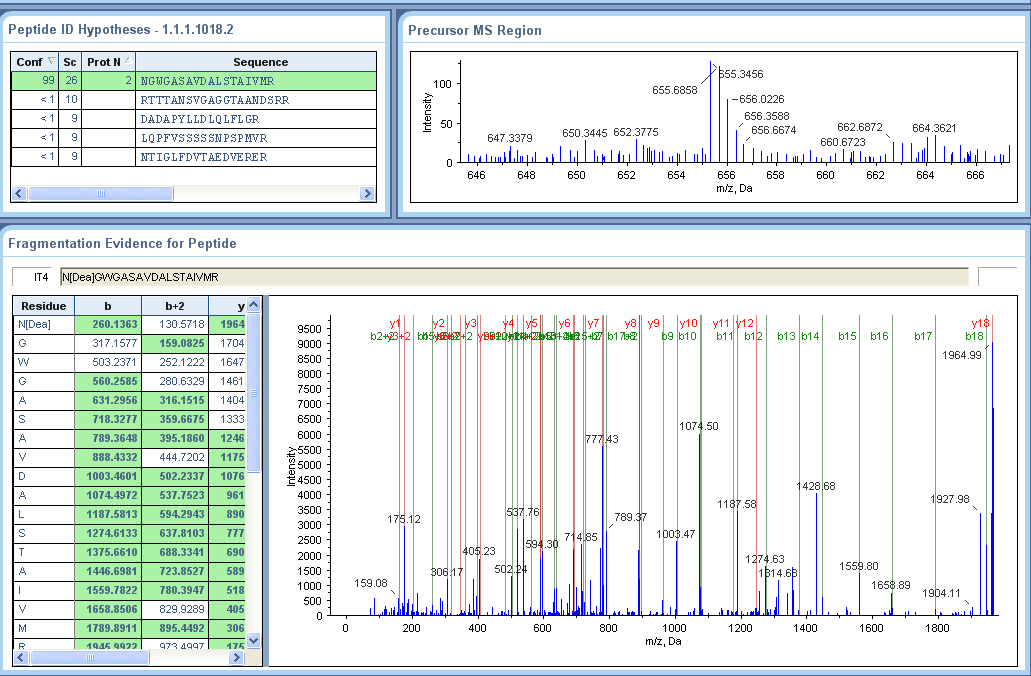


N#GWGASAVDALSTAIVMR


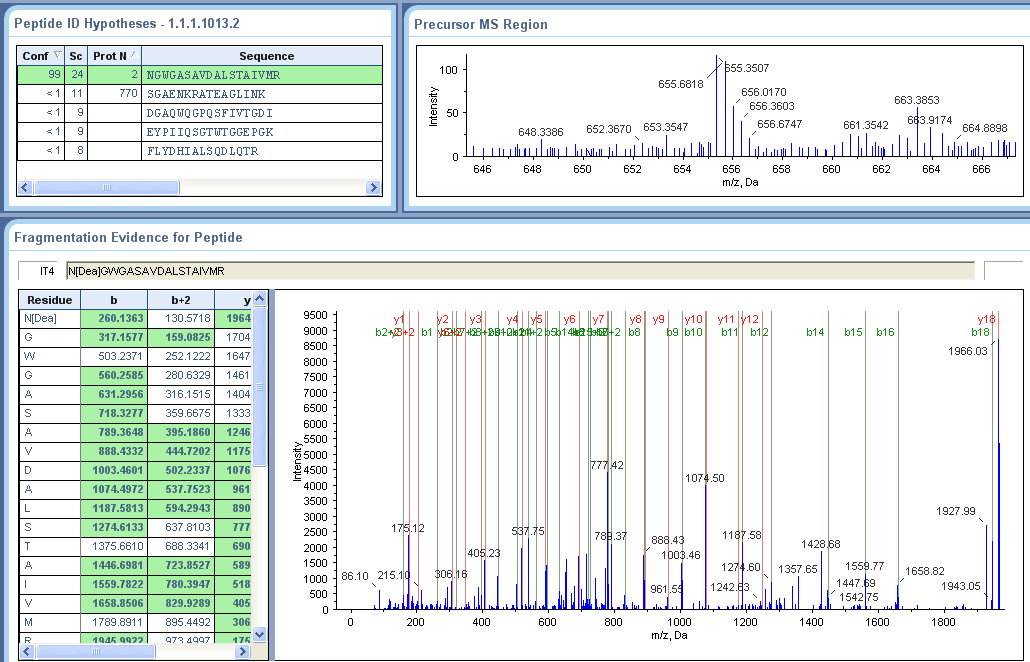


1. Afu1g14560| Aspergillus fumigatus alpha-mannosidase (494 aa)

N#ATIVSQILDHIAK
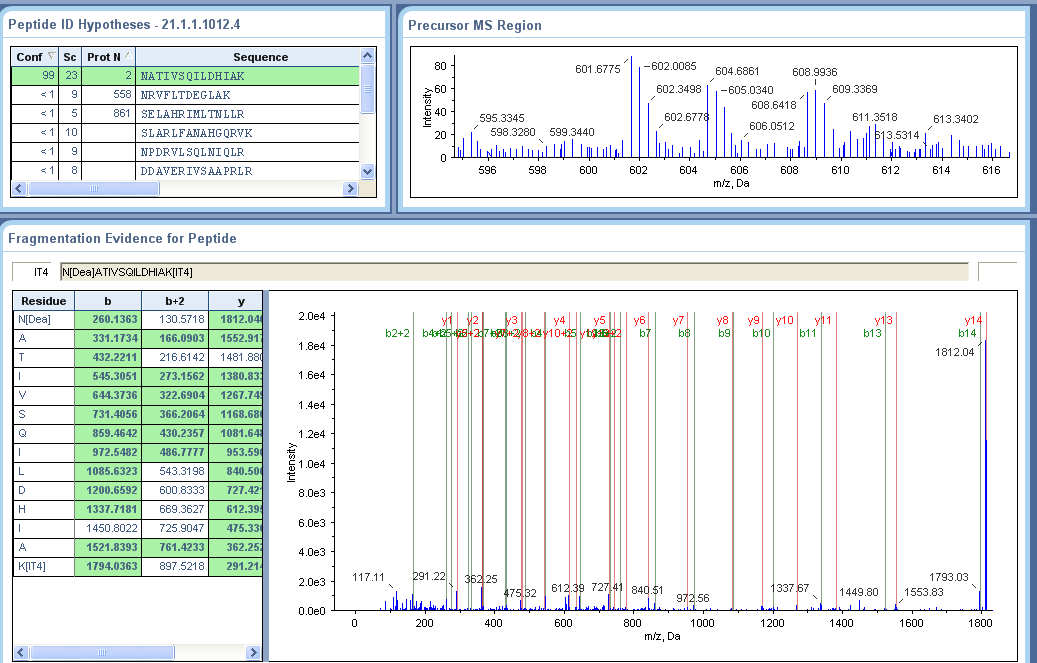


N#ATIVSQILDHIAK


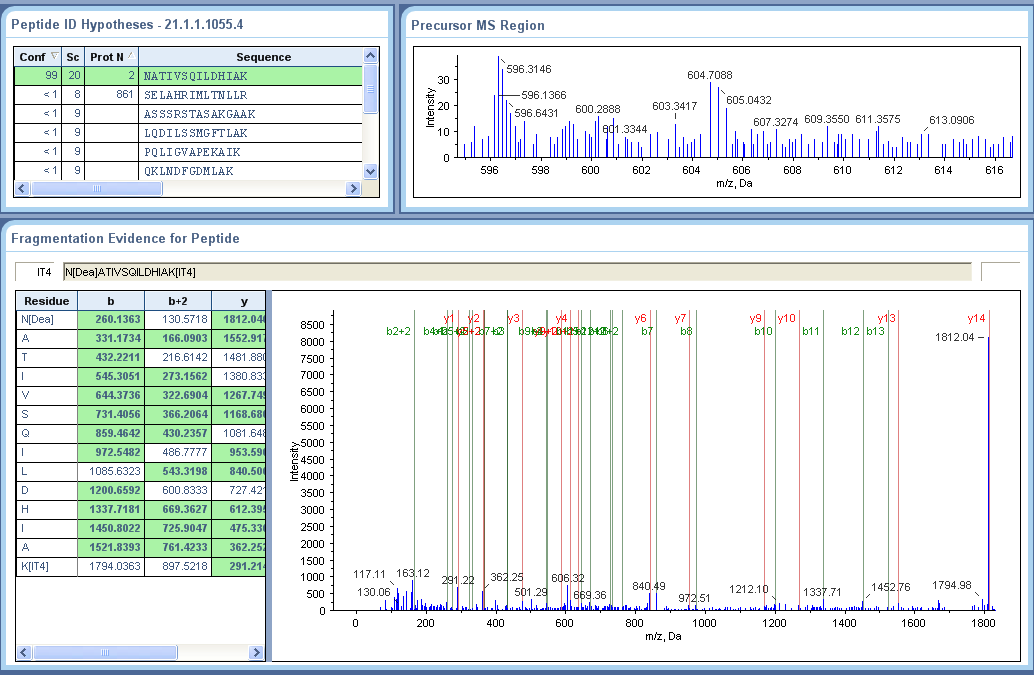


1. Afu1g14560| Aspergillus fumigatus alpha-mannosidase (494 aa)

N#GWGASAVDALSTAIVMR


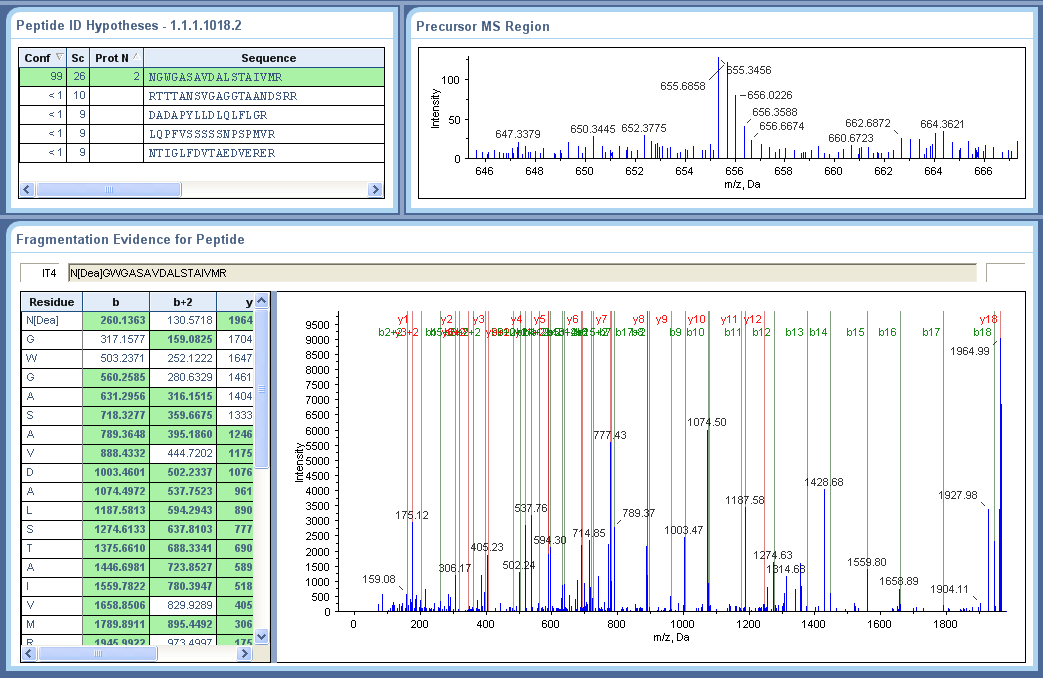


N#GWGASAVDALSTAIVMR
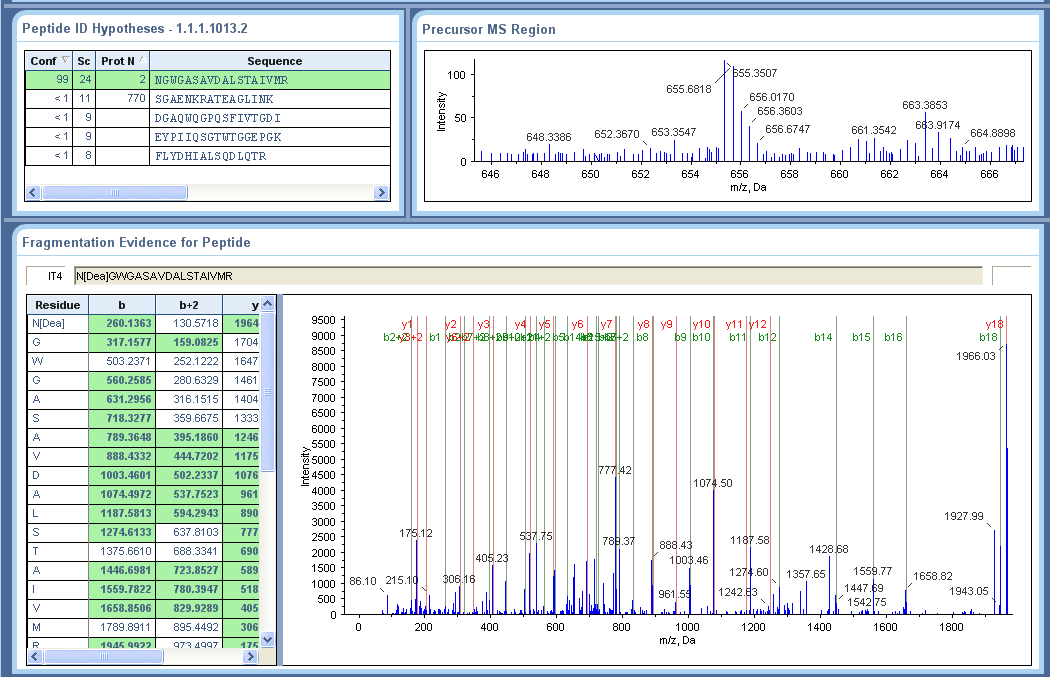


1. Afu3g00320 | Aspergillus fumigatus endo-1,4-beta-xylanase (XlnA), putative (229 aa)

GTVN#TDGGTYNIYTAVR


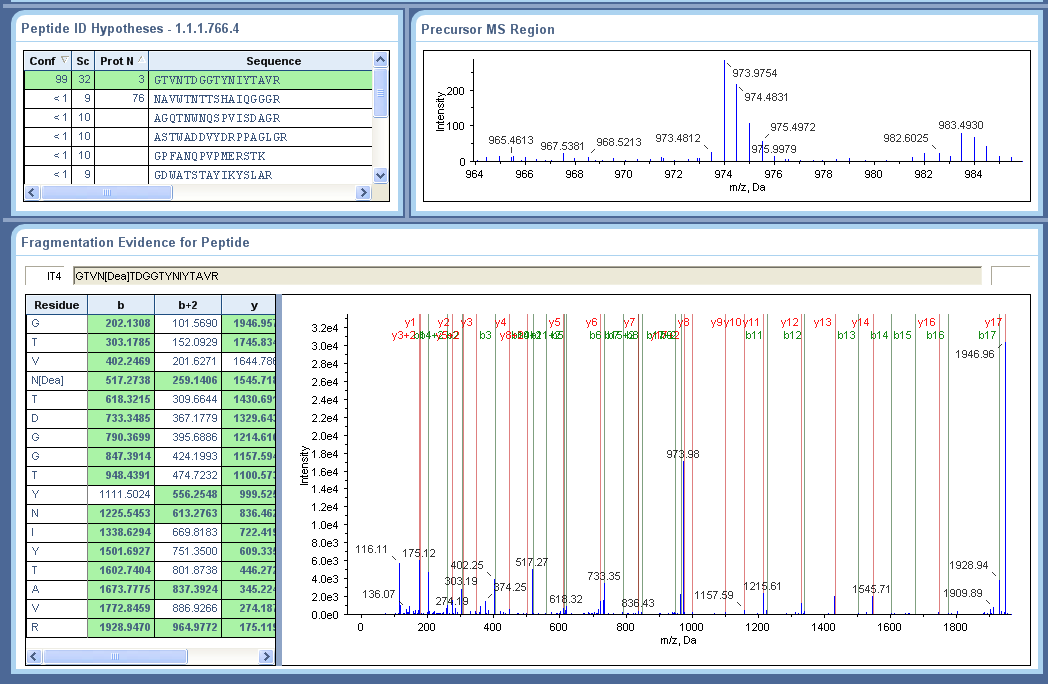


GTVN#TDGGTYNIYTAVR


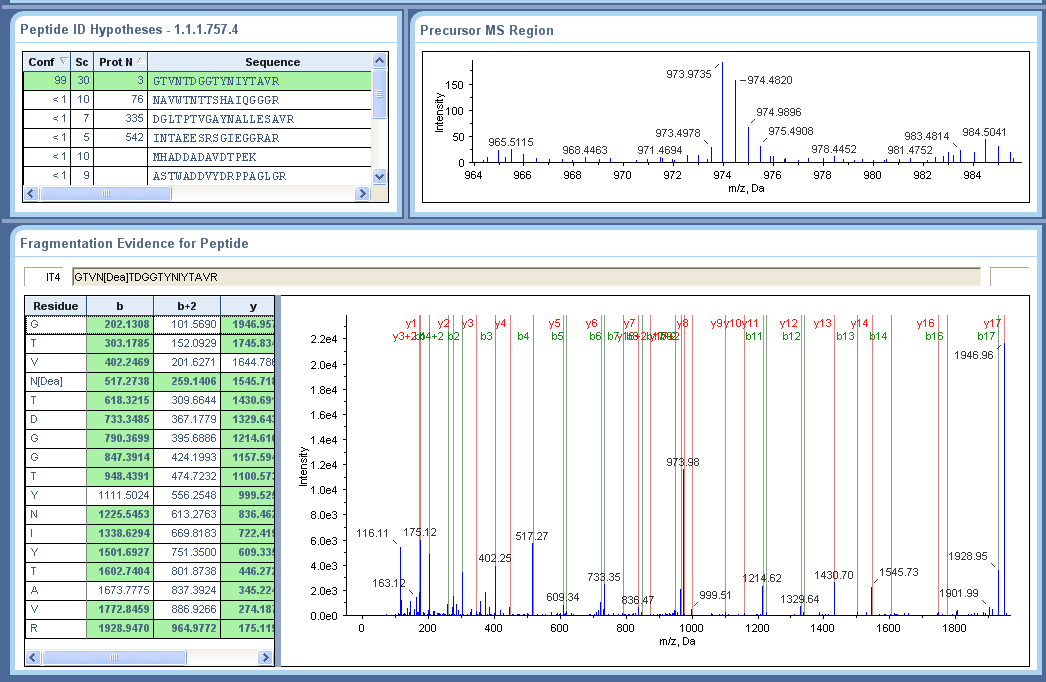


1. Afu3g00320| Aspergillus fumigatus endo-1,4-beta-xylanase (XlnA), putative (229 aa)

TFTQ#YWSVR


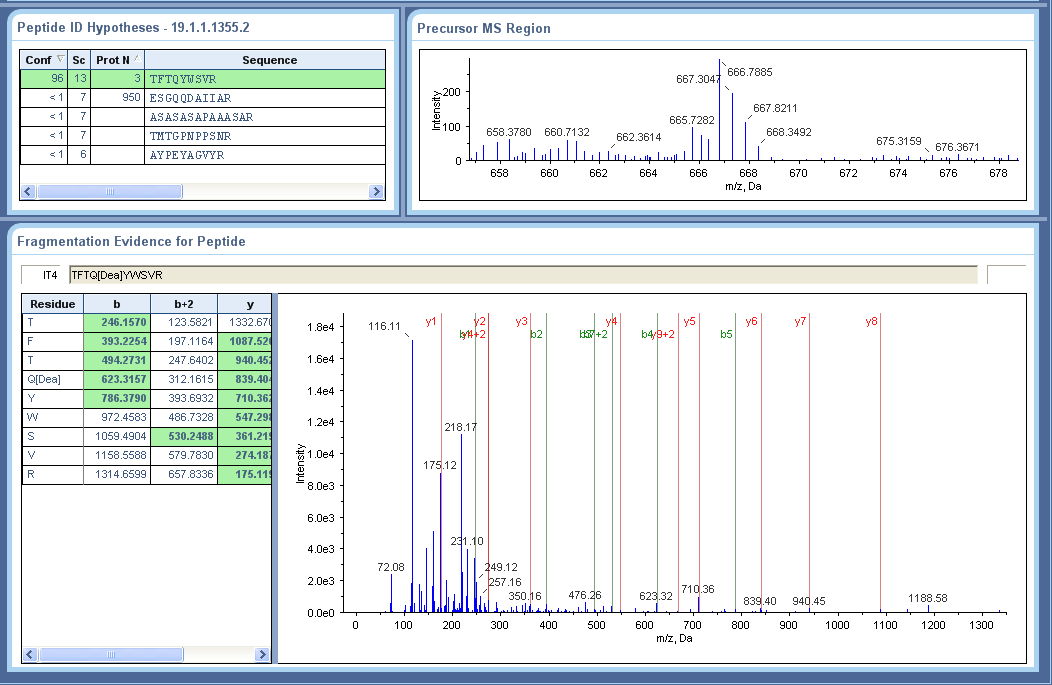


TFTQ#YWSVR


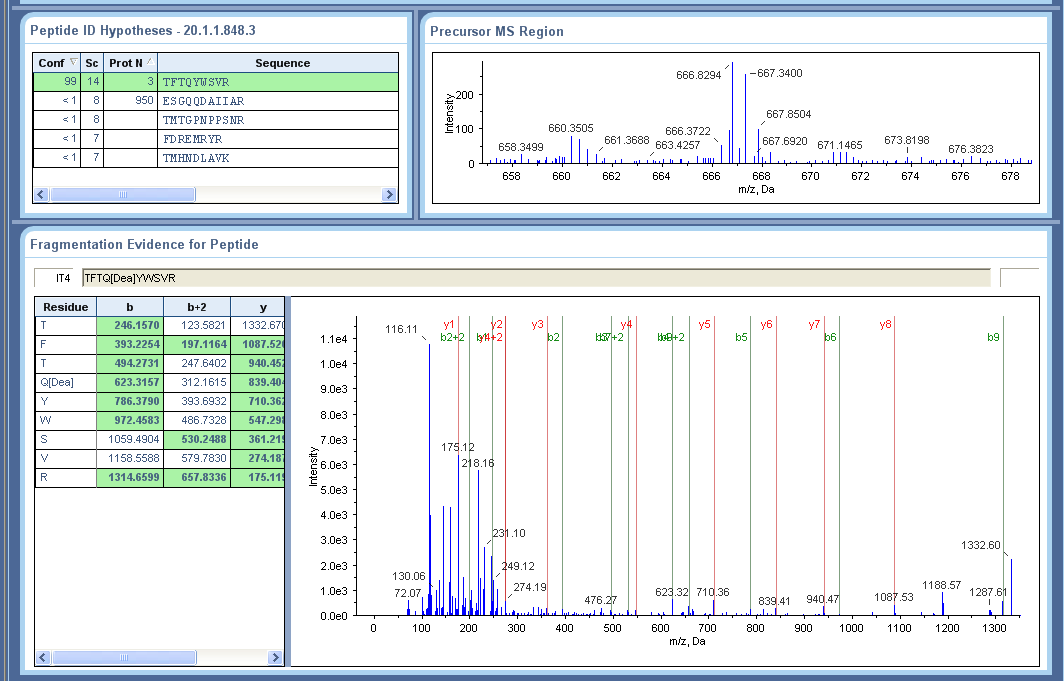


TFTQ#YWSVR


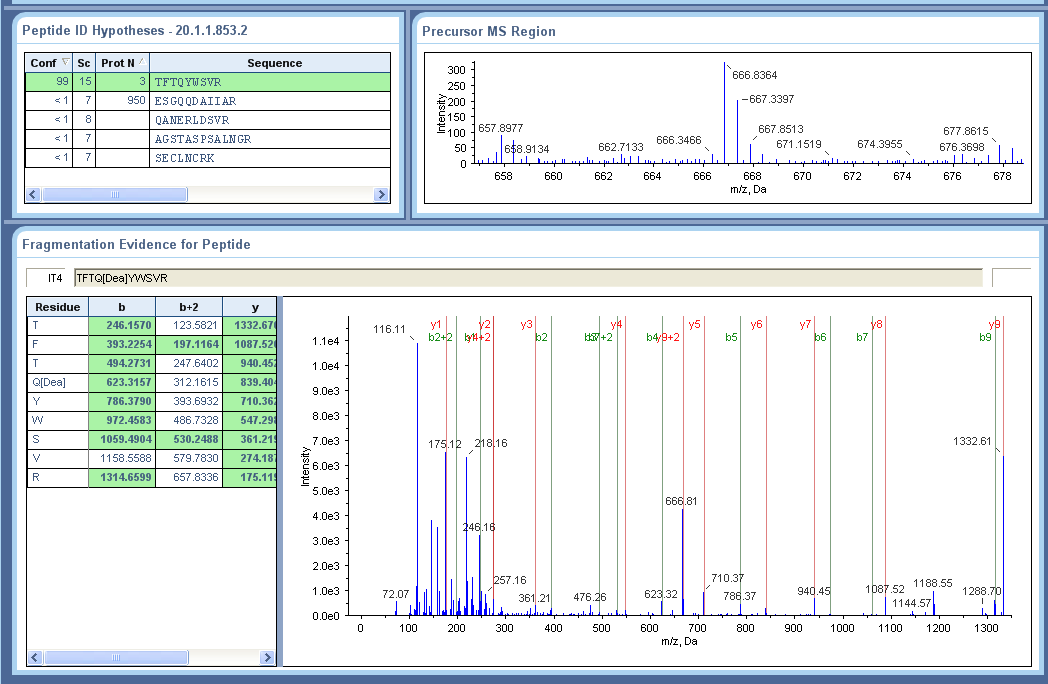


TFTQ#YWSVR


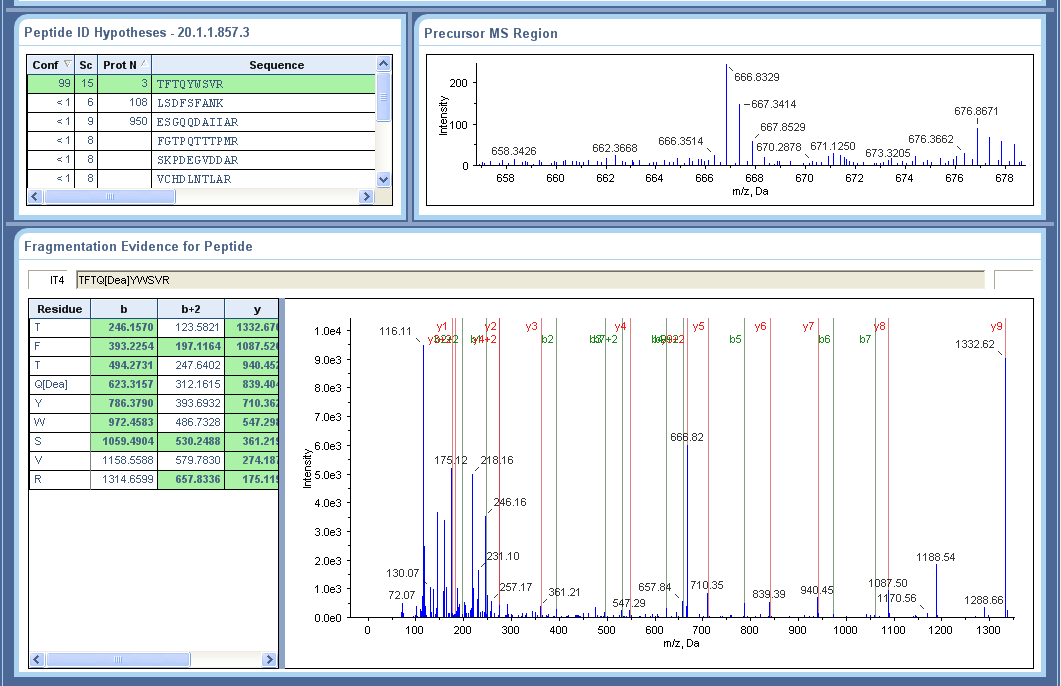


1. Afu2g00690| Aspergillus fumigatus glucan 1,4-alpha-glucosidase, putative (632 aa)

ATALIDFGNWLIDN#GYSSYAVNNIWPIVR


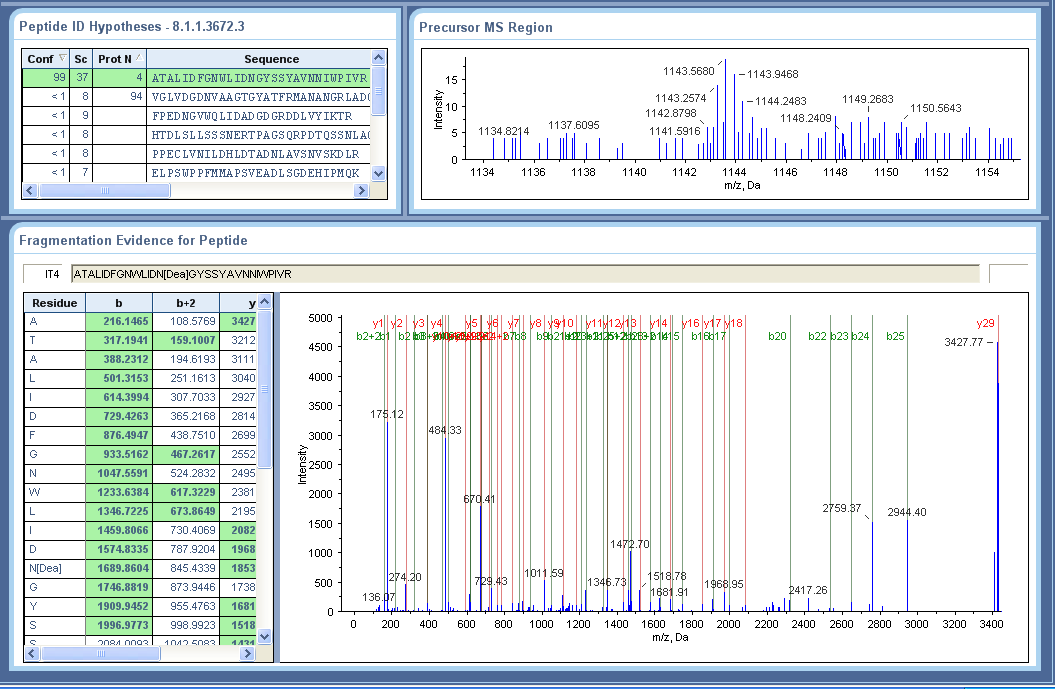


ATALIDFGNWLIDN#GYSSYAVNNIWPIVR


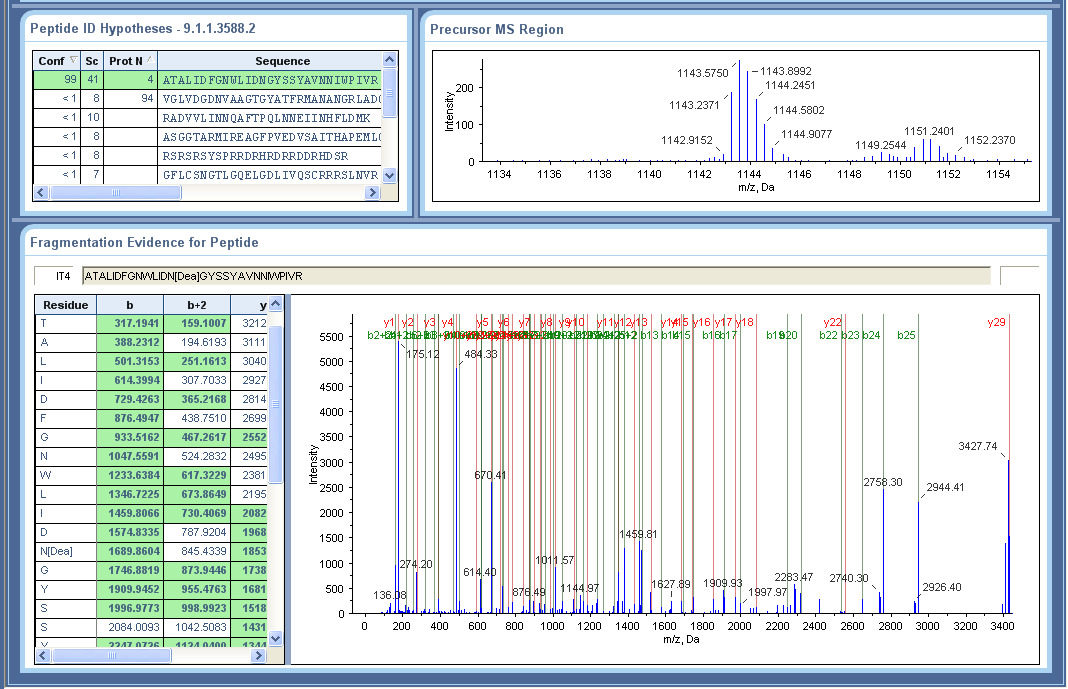


ATALIDFGN#WLIDNGYSSYAVNNIWPIVR


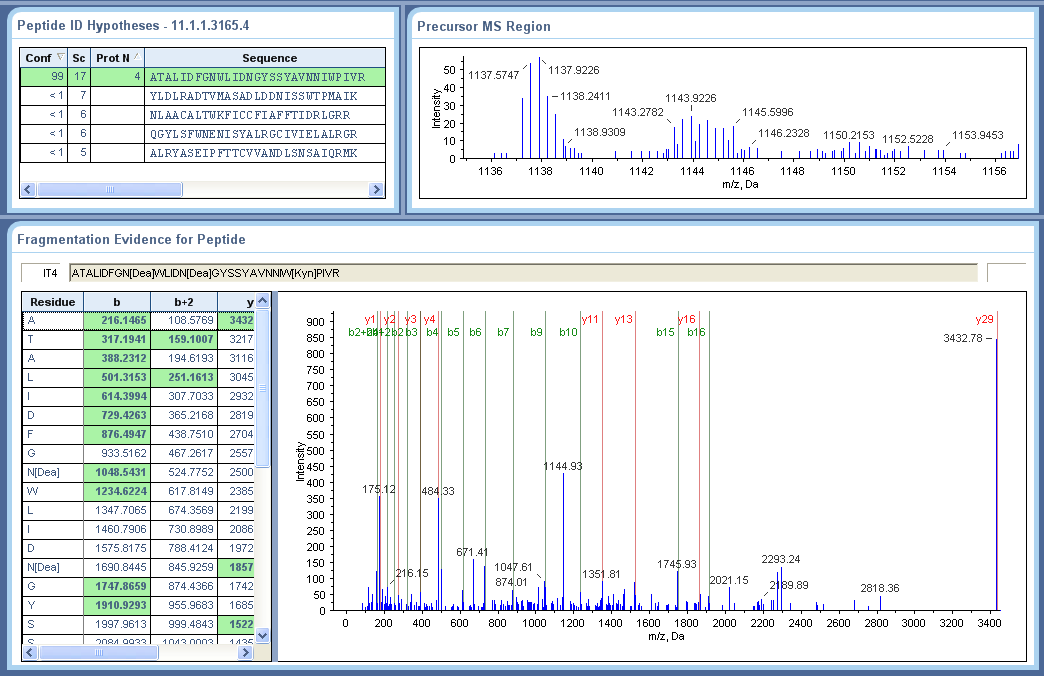


1. Afu2g00690| Aspergillus fumigatus glucan 1,4-alpha-glucosidase, putative (632 aa)


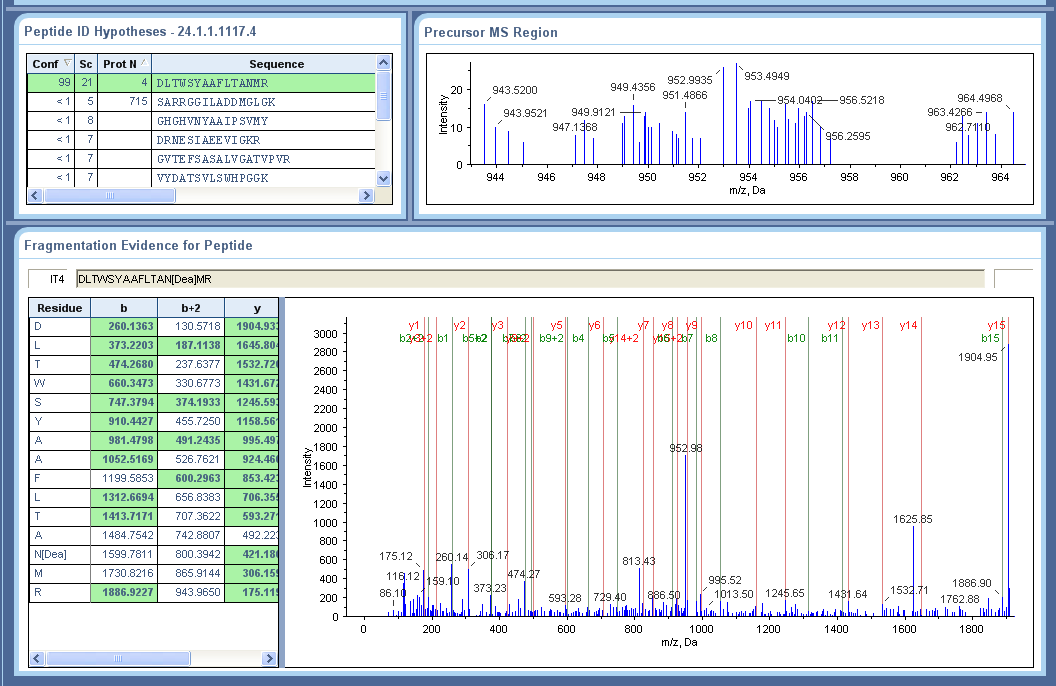


1. Afu4g09030| Aspergillus fumigatus aminopeptidase (954 aa)

LTFSGILN#DNMAGFYR


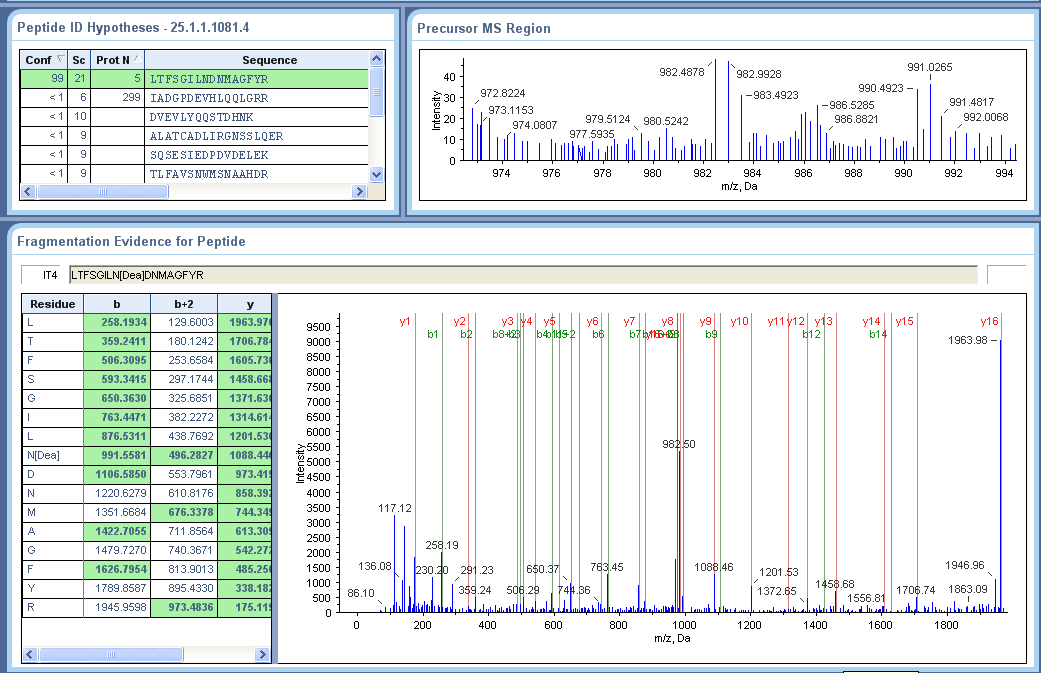


1. Afu3g02090| Aspergillus fumigatus beta-xylosidase (772 aa)

HFAAYDLEDWN#GVVR


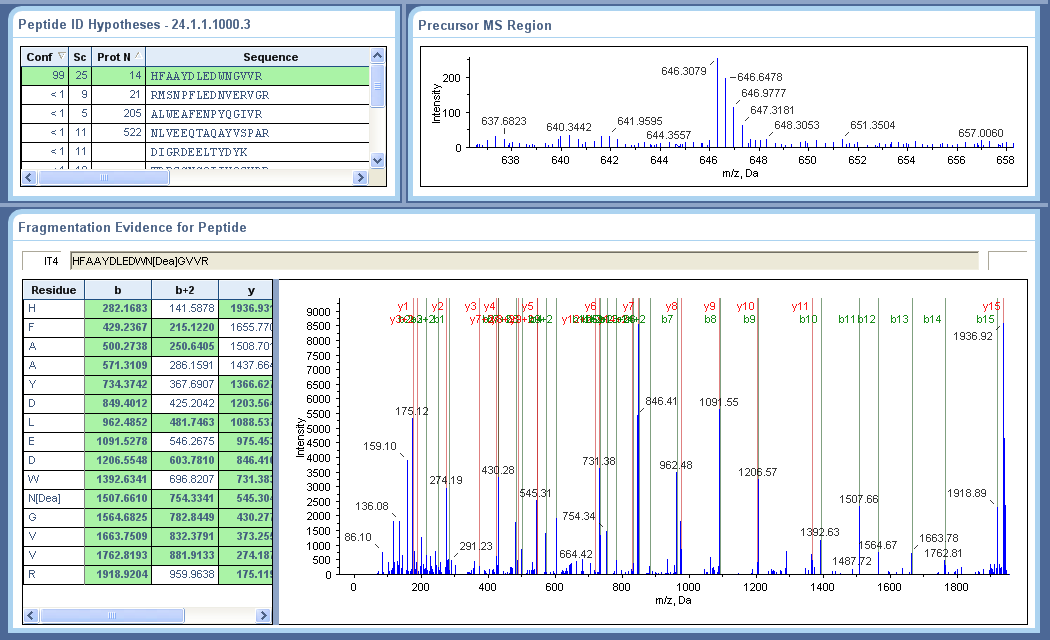


HFAAYDLEDWN#GVVR


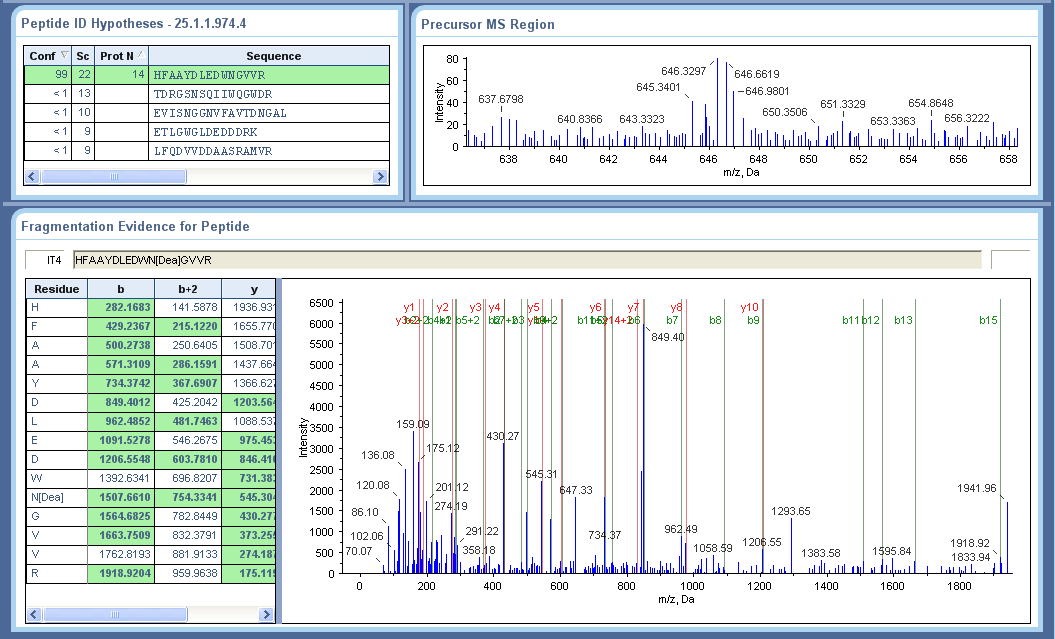


1. Afu6g03150| Aspergillus fumigatus hypothetical protein (431 aa)

DQTQYLQ#MWGR


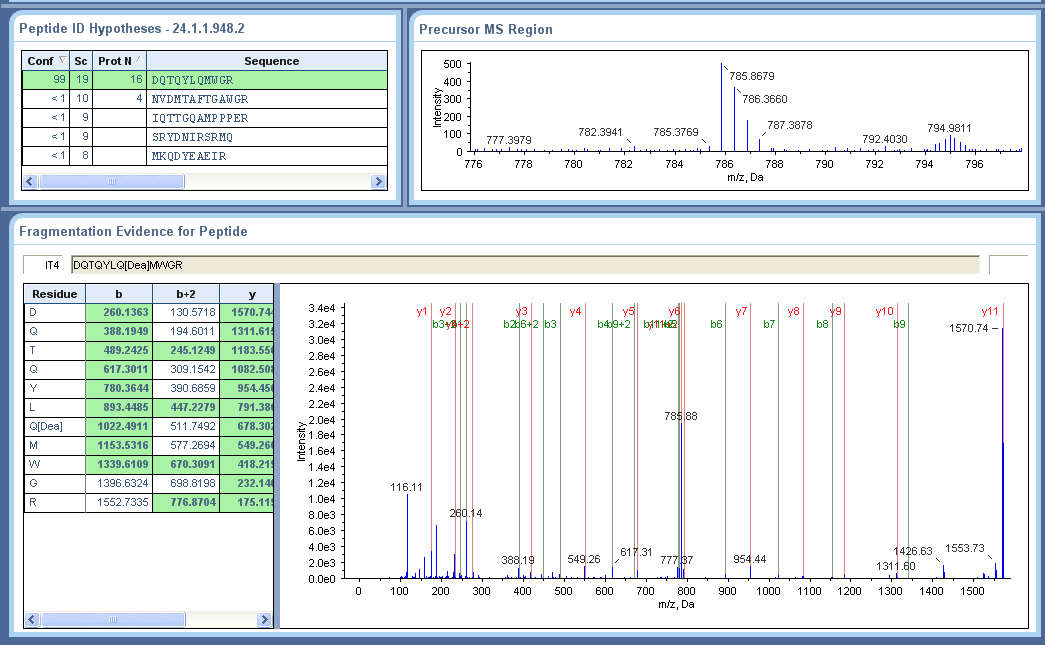


DQTQYLQ#MWGR


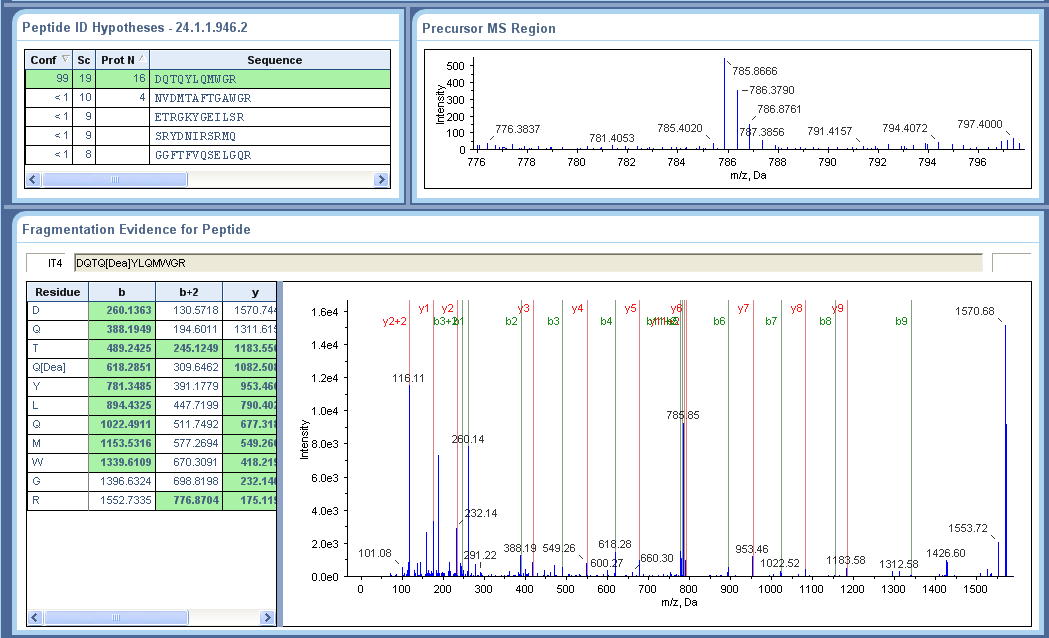


DQTQYLQ#MWGR


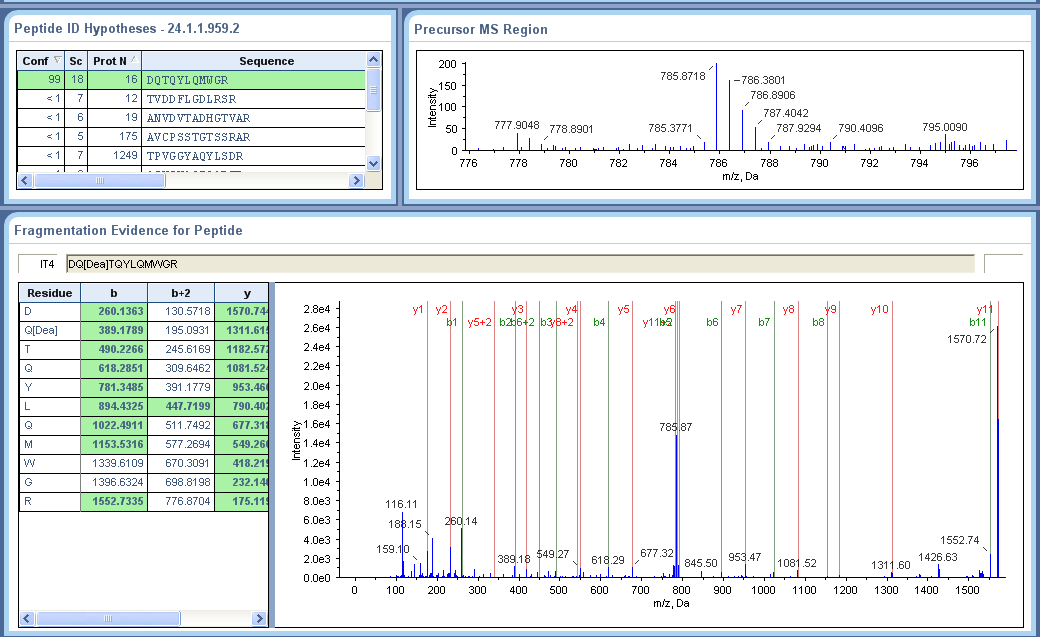


1. Afu2g03980| Aspergillus fumigatus alpha-1,3-glucanase, putative (497 aa)

WQEILN#LGPR


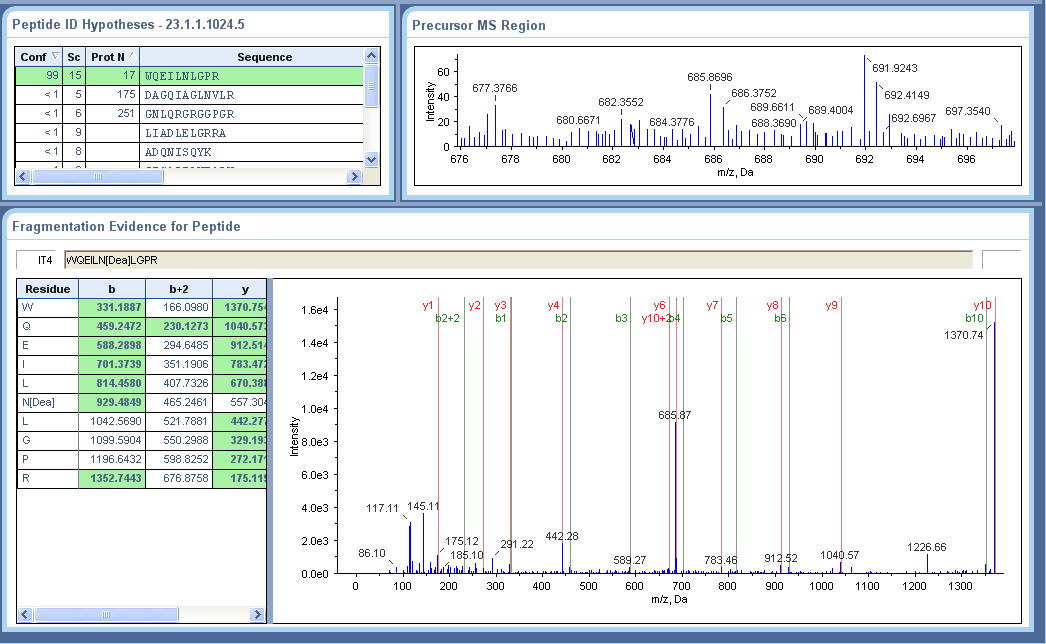


1. Afu7g06150| Aspergillus fumigatus endoglucanase, putative (235 aa)

LVSQISSIPTTVQWSYDNTN#TR


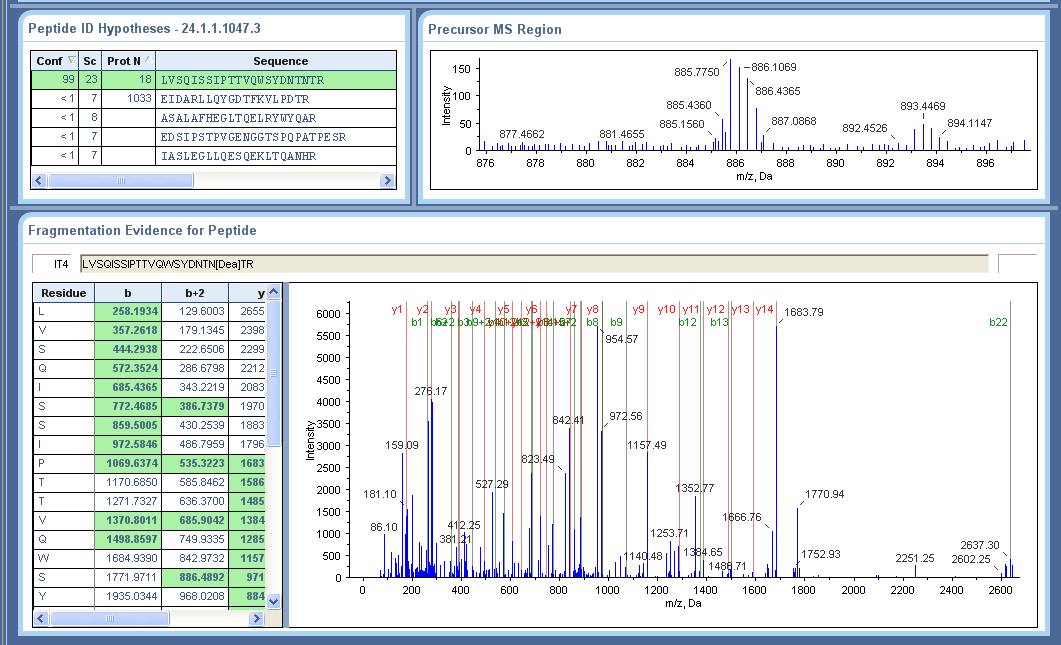


LVSQISSIPTTVQ#WSYDNTNTR


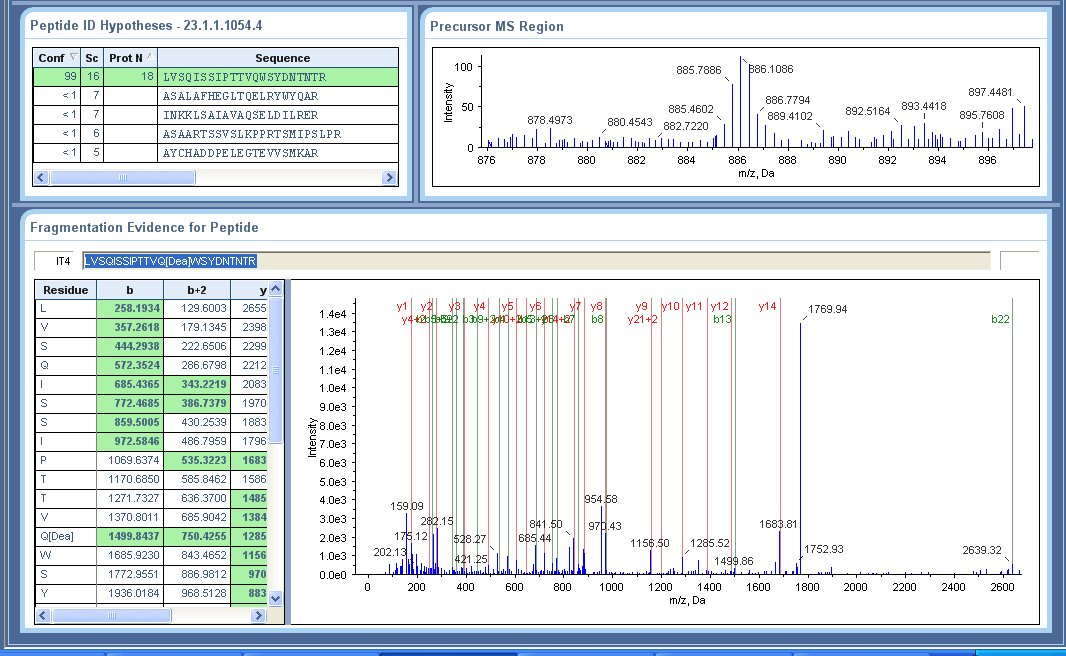


1. Afu7g06150| Aspergillus fumigatus endoglucanase, putative (235 aa)

N#HGYPASSQYLINMQFGTEPFTGGPATLR


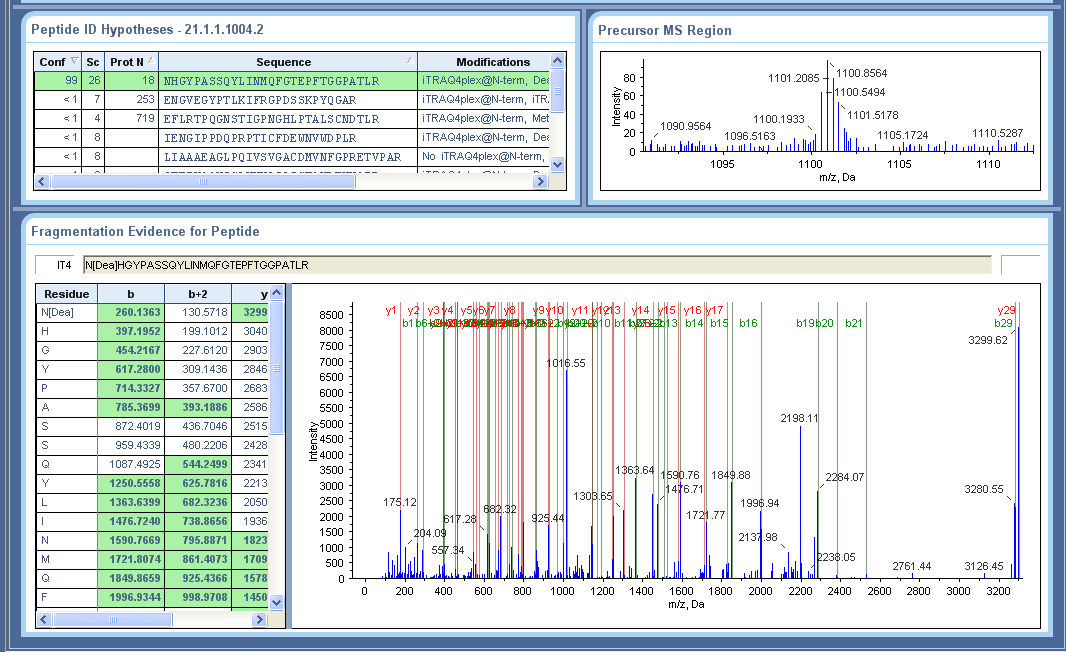


N#HGYPASSQYLINMQFGTEPFTGGPATLR


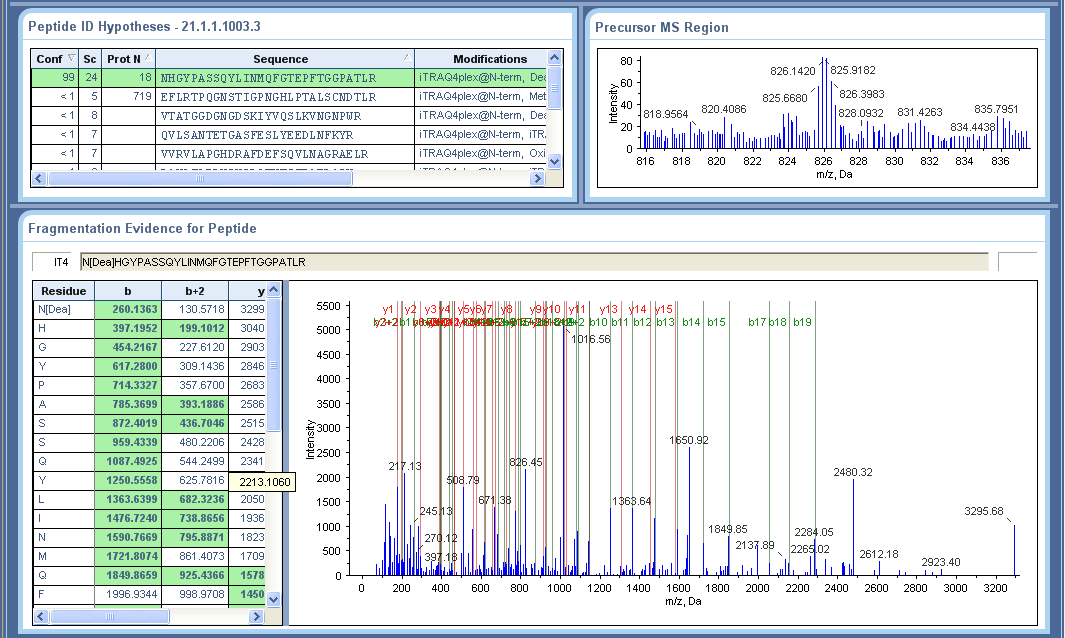


1. Afu7g06150| Aspergillus fumigatus endoglucanase, putative (235 aa)

VSQ#WTASVN


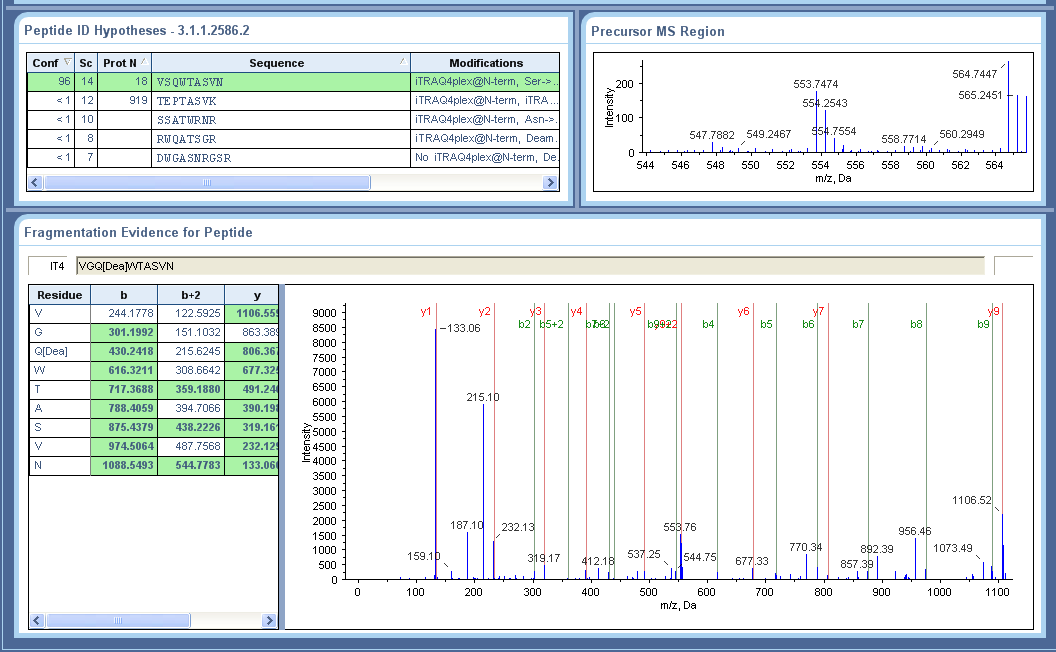


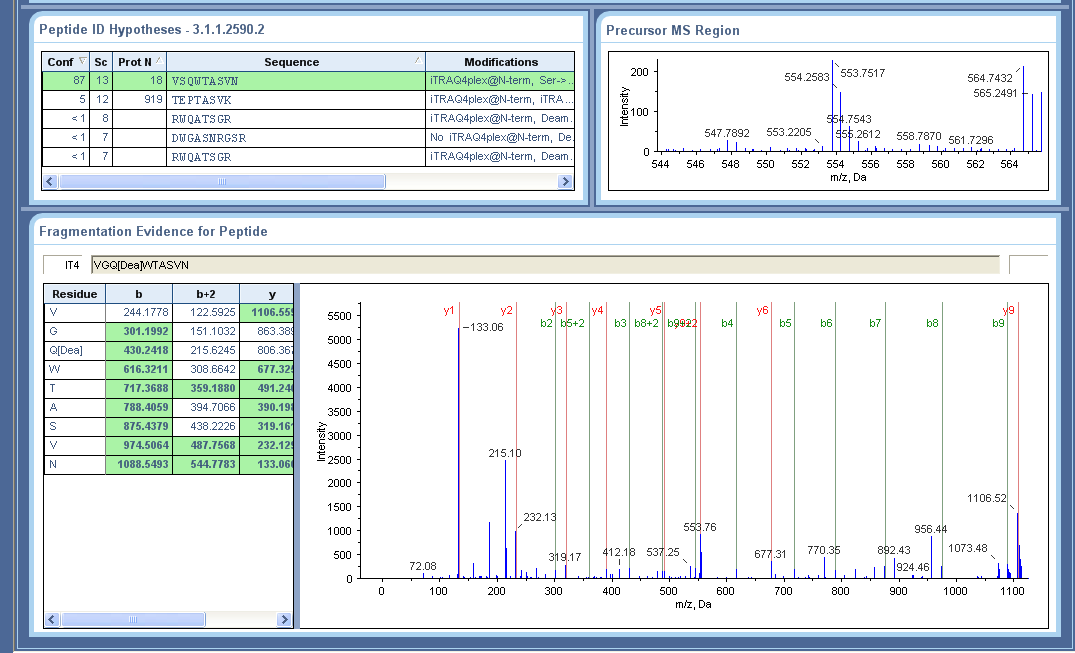


1. Afu7g05610| Aspergillus fumigatus glucanase, putative (471 aa)

YSLLGEQ#LR


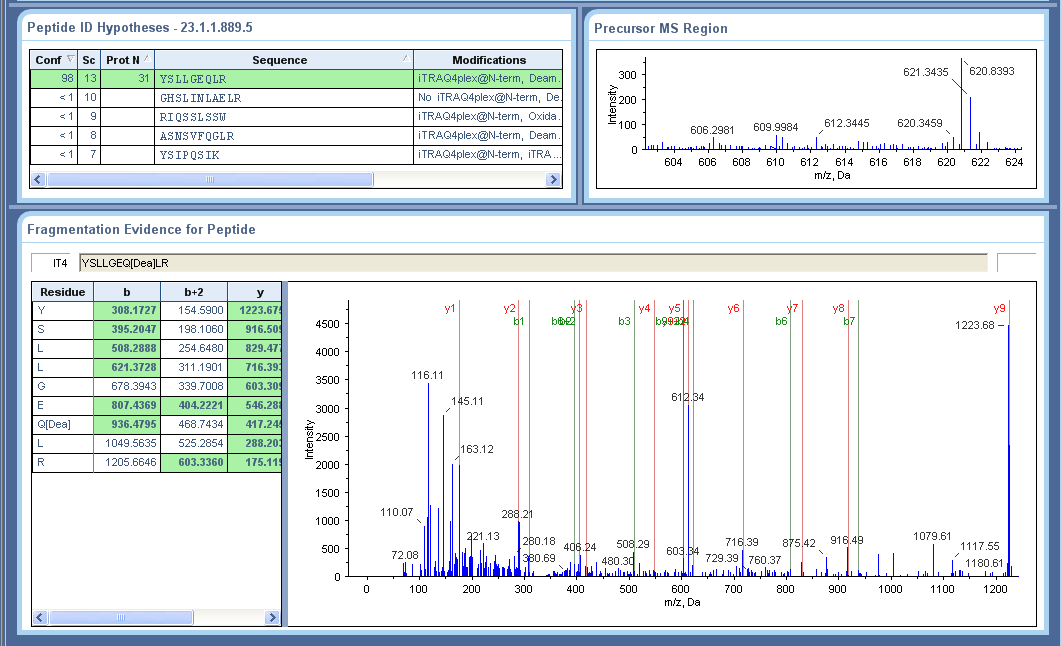


1. Afu4g09480| Aspergillus fumigatus extracellular endo-1,4-beta-xylanase, putative (325 aa)

AAYN#AIIAAL


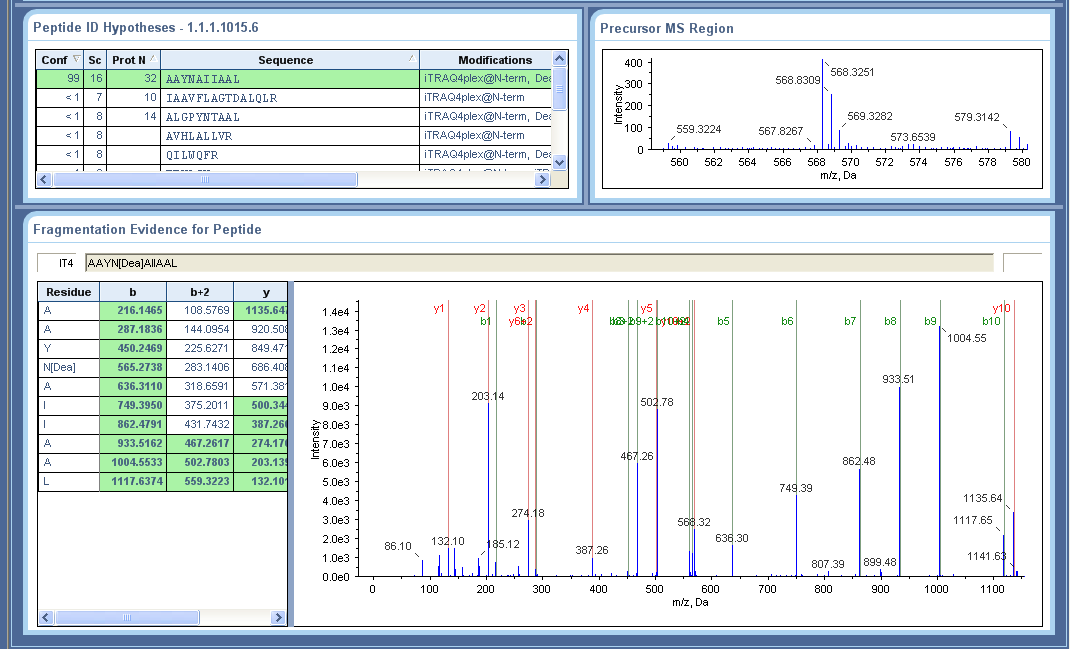


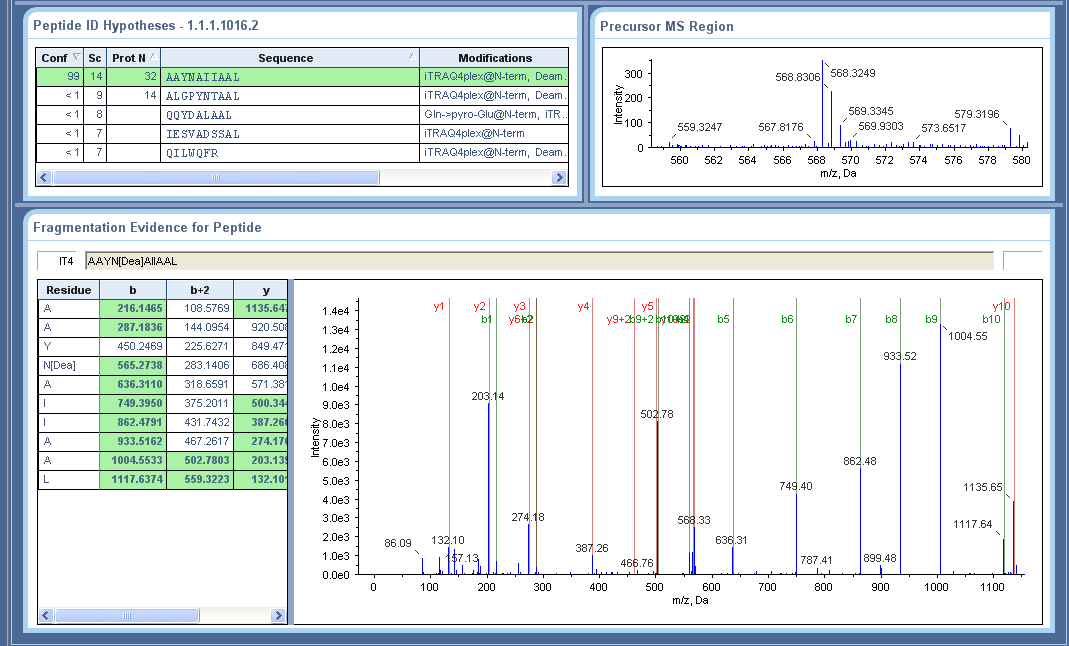


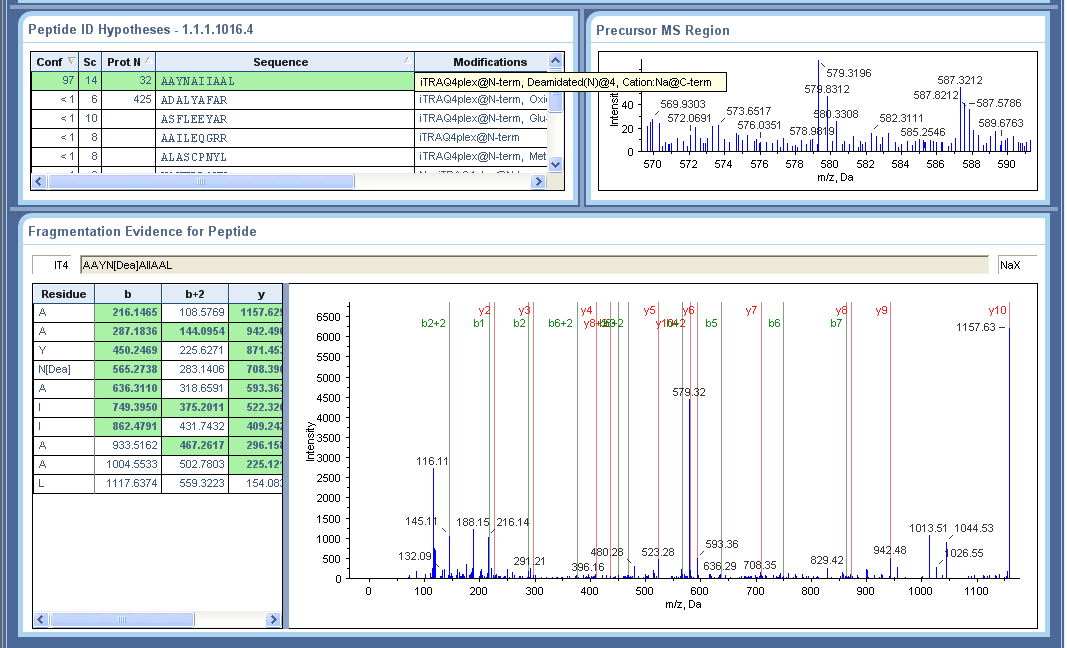


1. Afu1g10790| Aspergillus fumigatus alpha-1,2-mannosidase family protein, putative (840 aa)

SIN#GYPLPGGAFVR


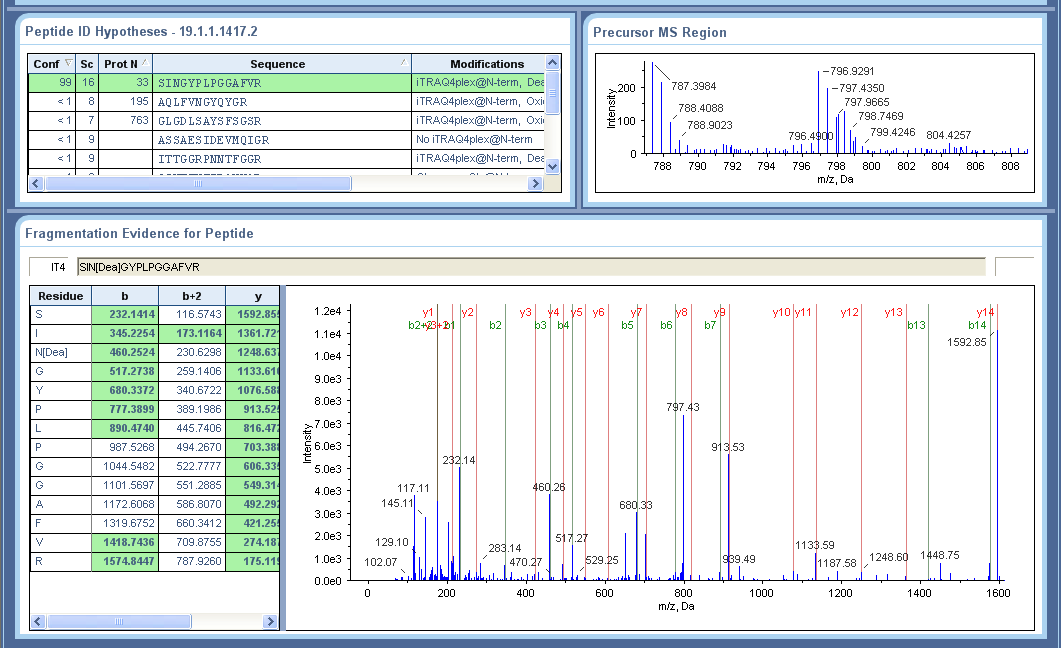


SIN#GYPLPGGAFVR


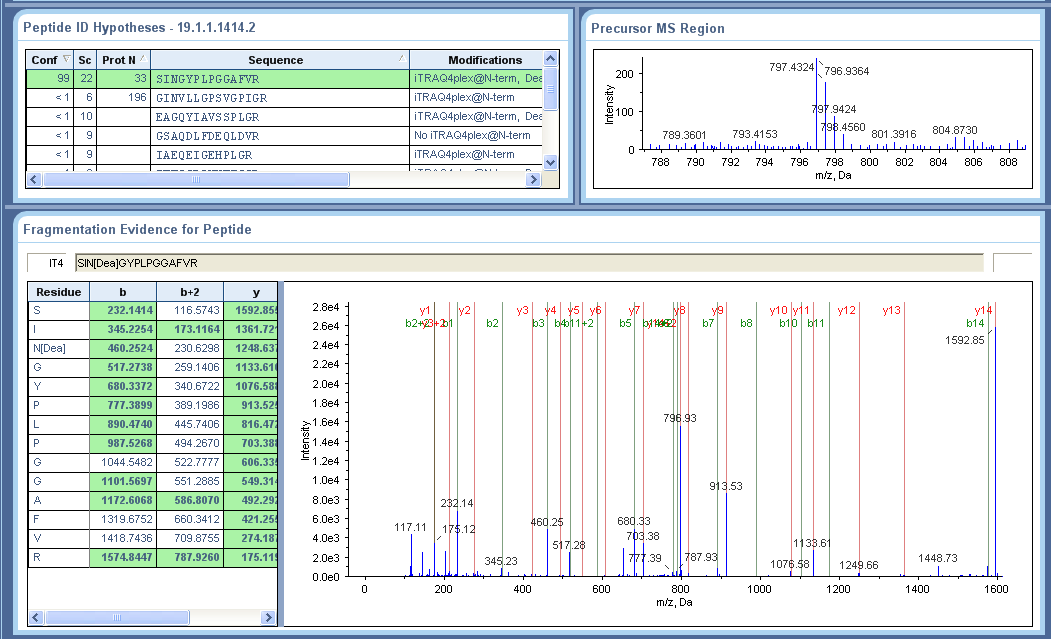


SIN#GYPLPGGAFVR


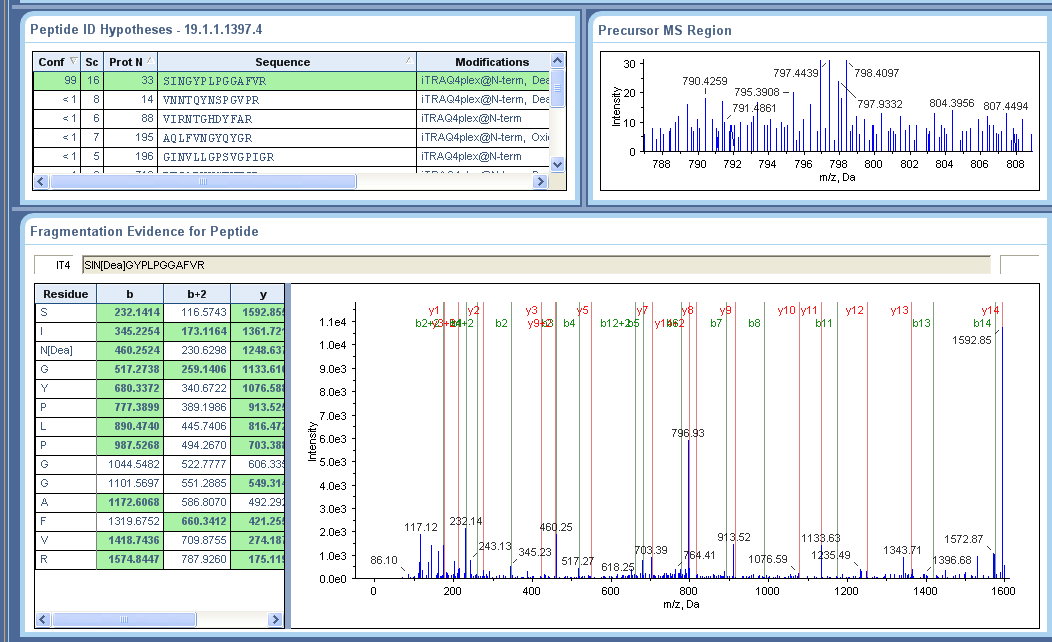


SIN#GYPLPGGAFVR


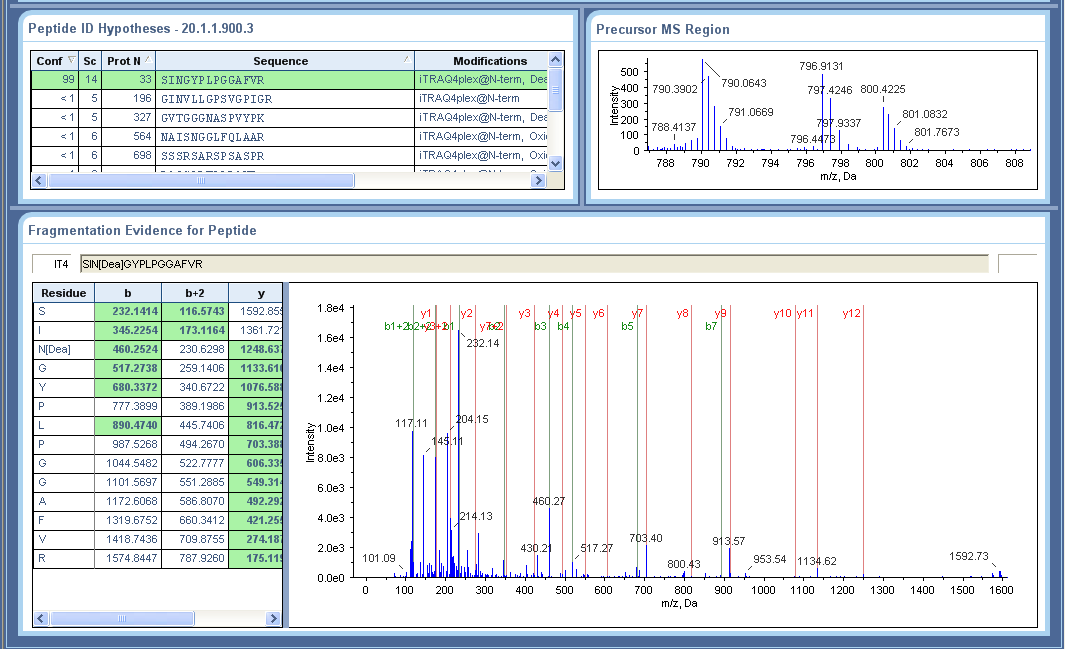


1. Afu6g13270| Aspergillus fumigatus exo-beta-1,3-glucanase, putative (805 aa)

ATPGFTGLGLIDGDQ#YQGDGNQGWISTNVFFR


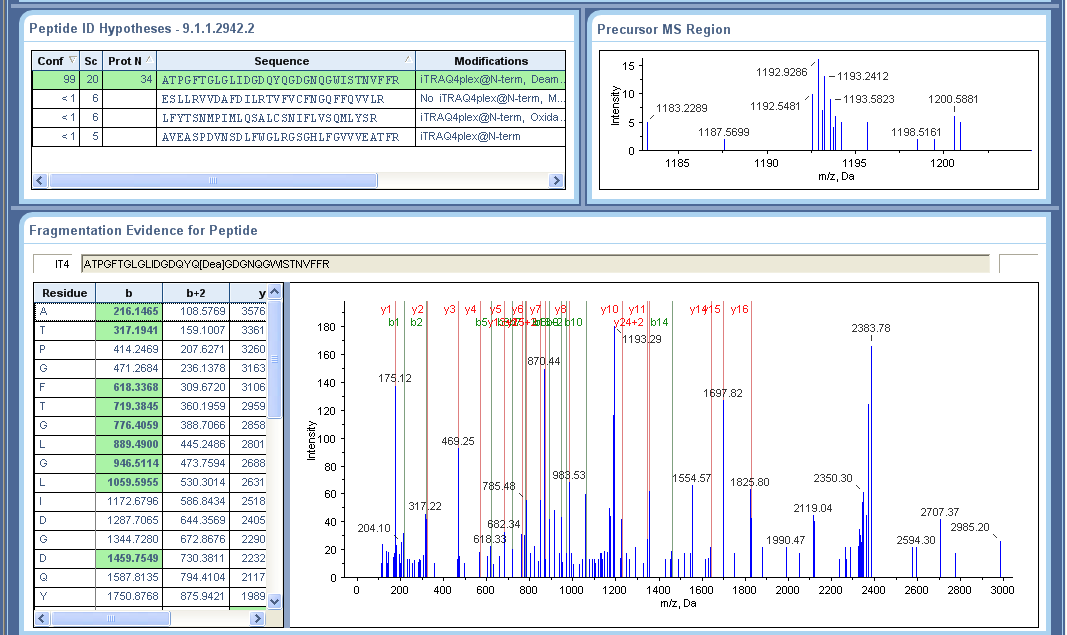


ATPGFTGLGLIDGDQ#YQGDGNQGWISTNVFFR


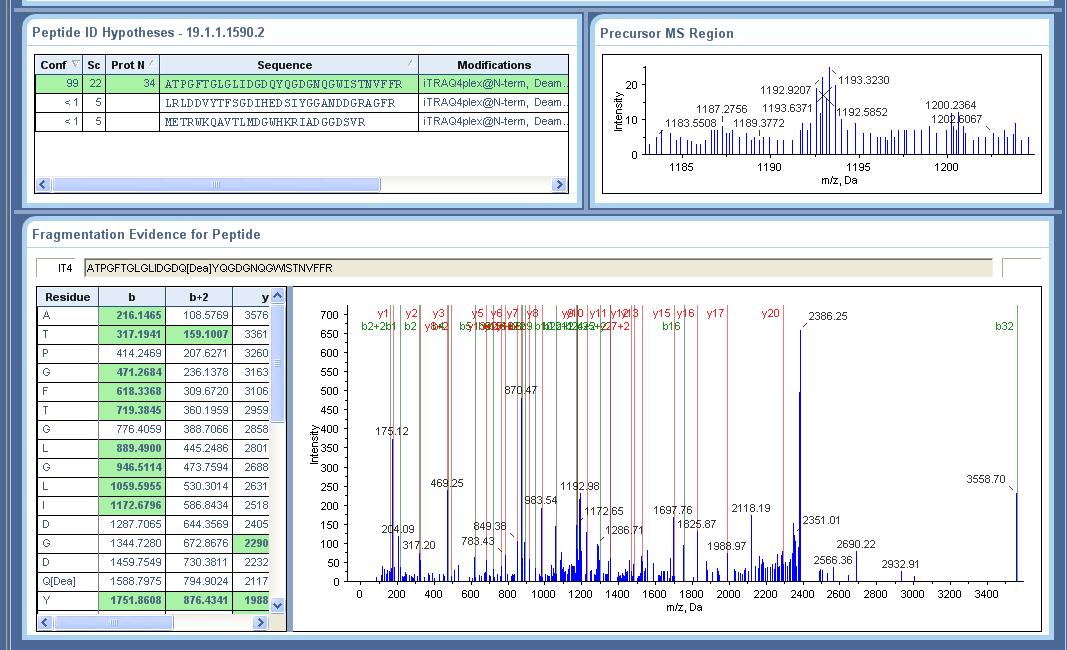


1. Afu1g04260| Aspergillus fumigatus endo-1,3-beta-glucanase Engl1 (975 aa)

GN#LMLGILR


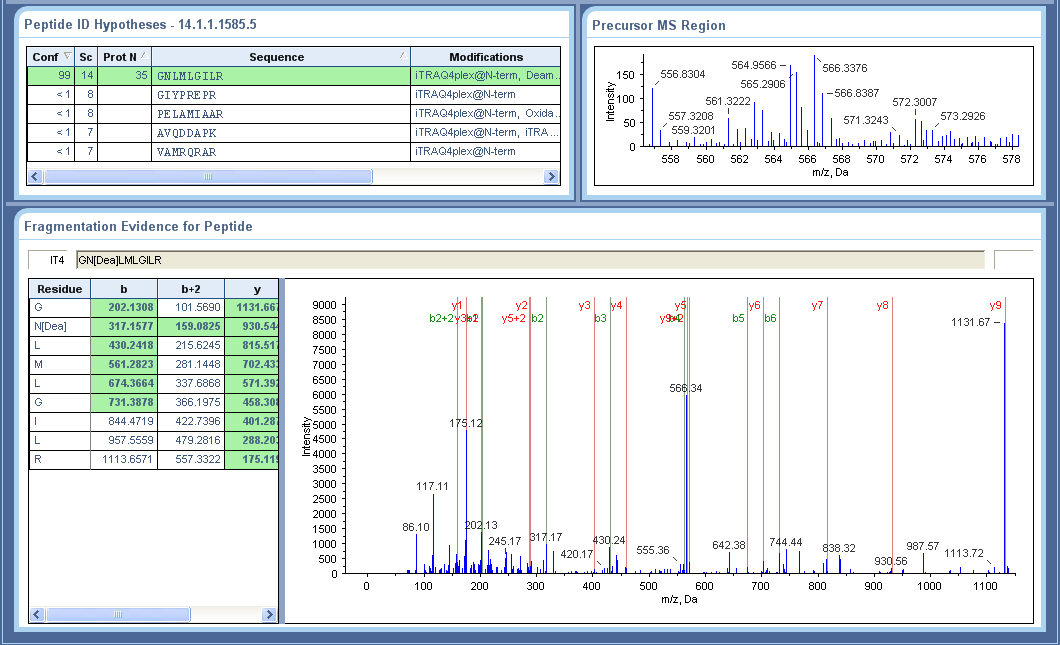


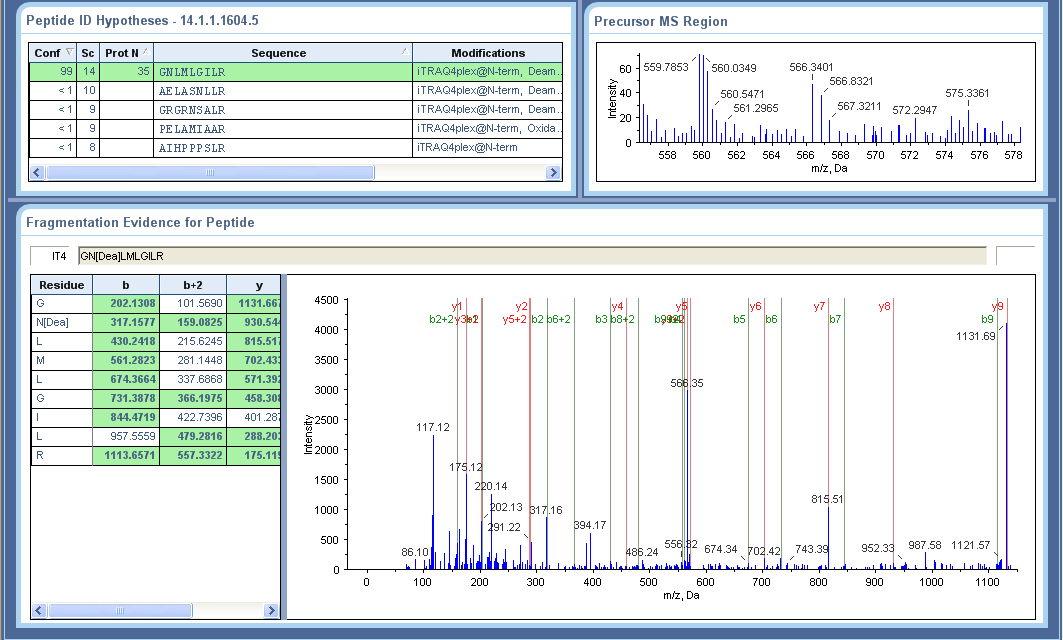


1. Afu8g07120| Aspergillus fumigatus beta-1,6-glucanase, putative (489 aa)

FMPPGAIVLN#GSGSYTYSGGGGIQSVASLNPDGTR


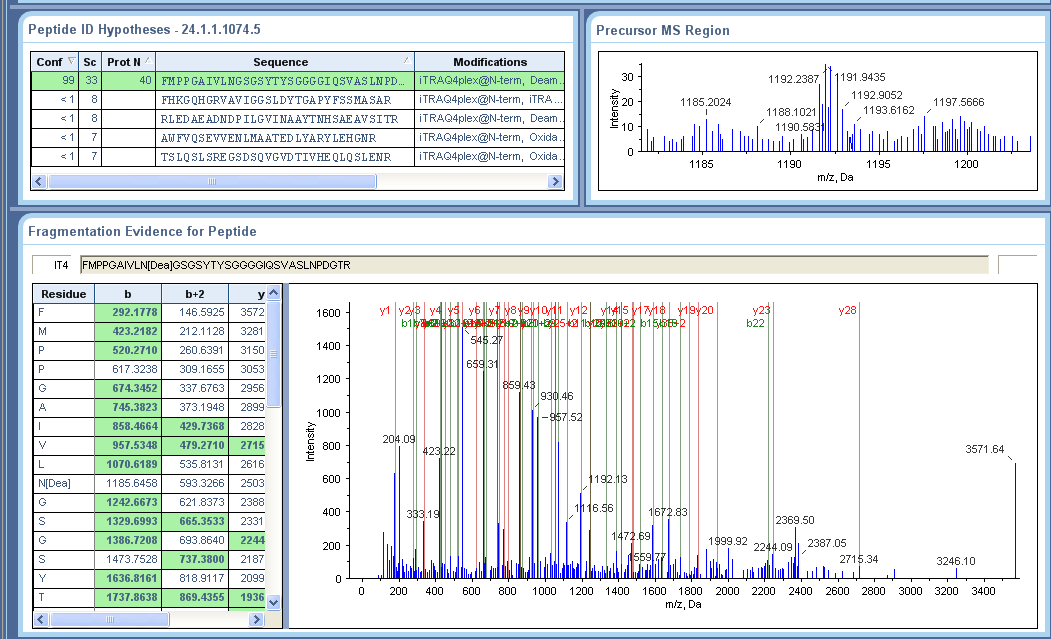


1. Afu8g07120| Aspergillus fumigatus beta-1,6-glucanase, putative (489 aa)


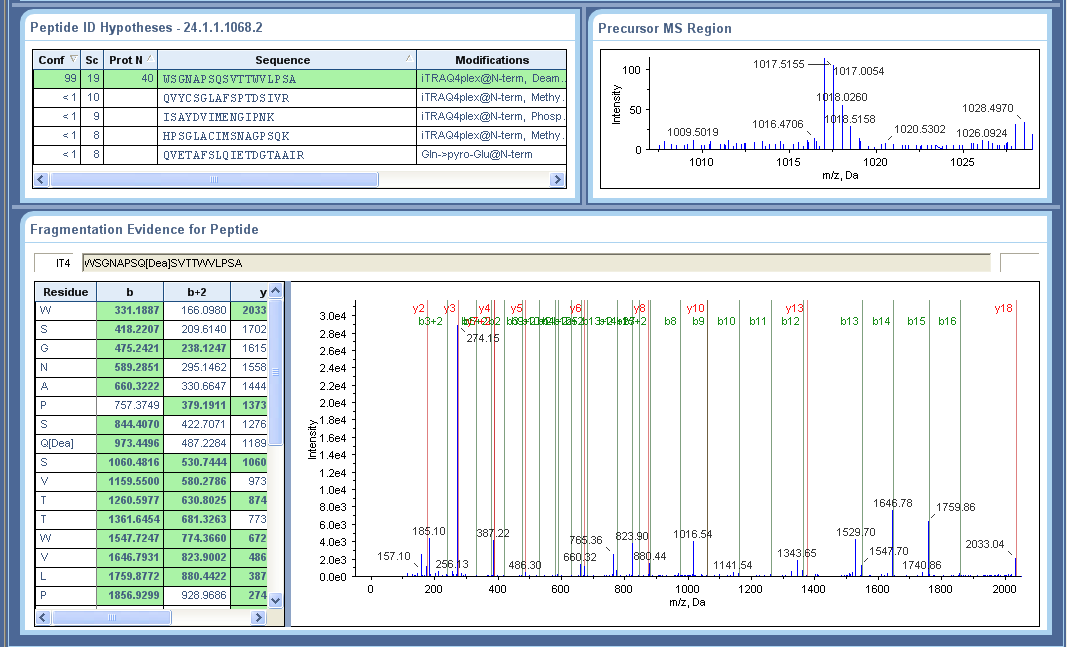


1. Afu4g11800| Aspergillus fumigatus alkaline serine protease Alp1 (404 aa)

ASFSNYGSVVDIFAPGQDILSAWIGSTTATN#TISGTSMATPH

**
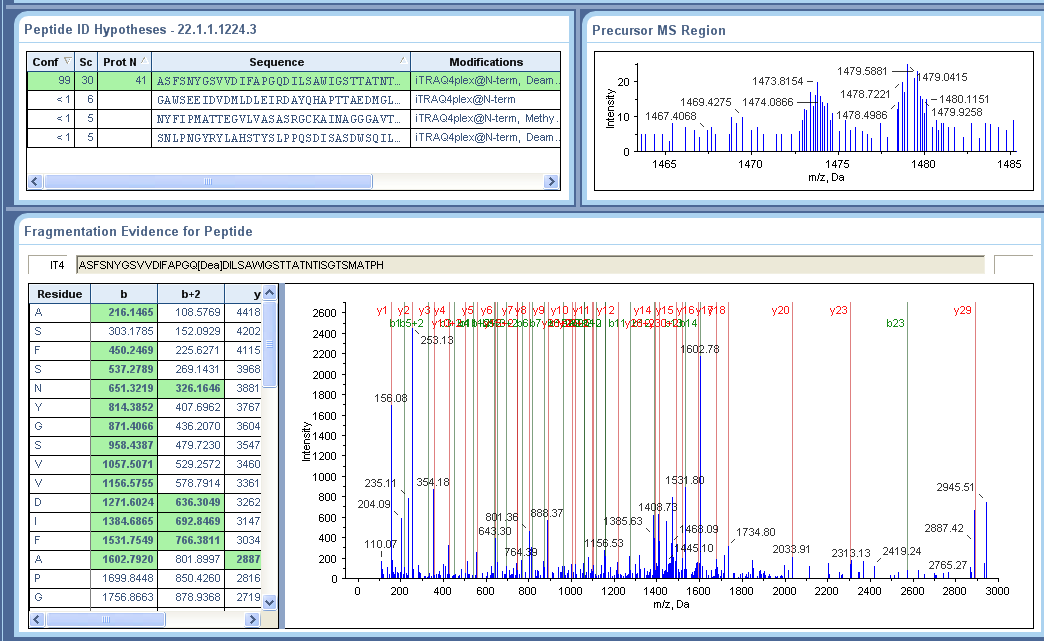
**

1. Afu3g02600| Aspergillus fumigatus hypothetical protein (281 aa)

AQAALGSN#ALVSSTVVAASR

**
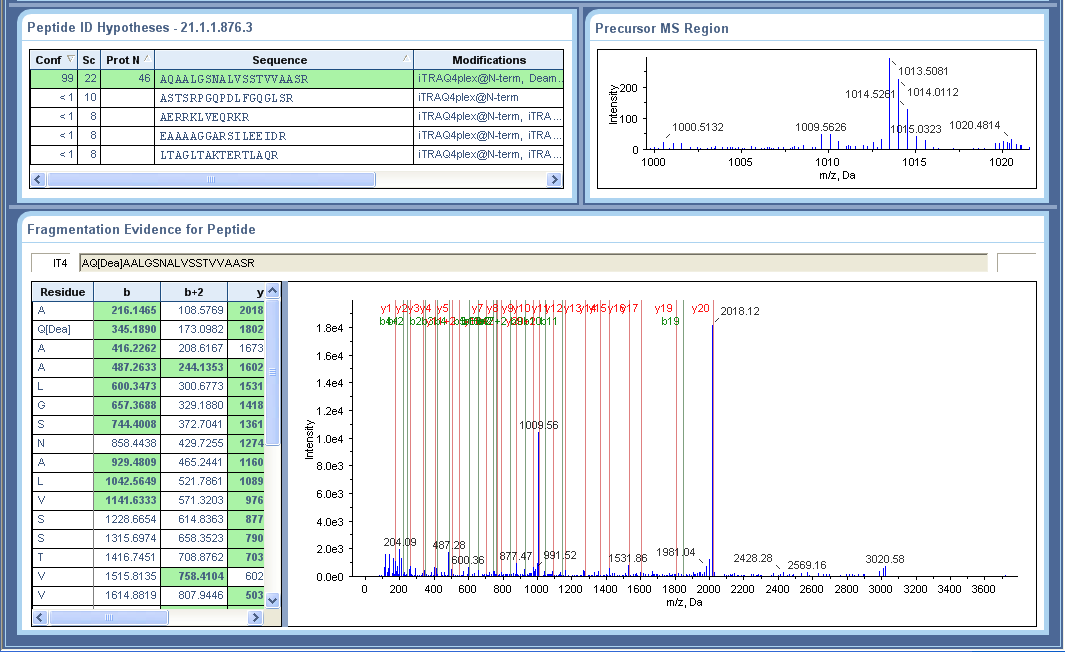
**

1. Afu6g14620| Aspergillus fumigatus alpha-L-arabinofuranosidase (507 aa)

GANAASGSLSTFYN#GAR

**
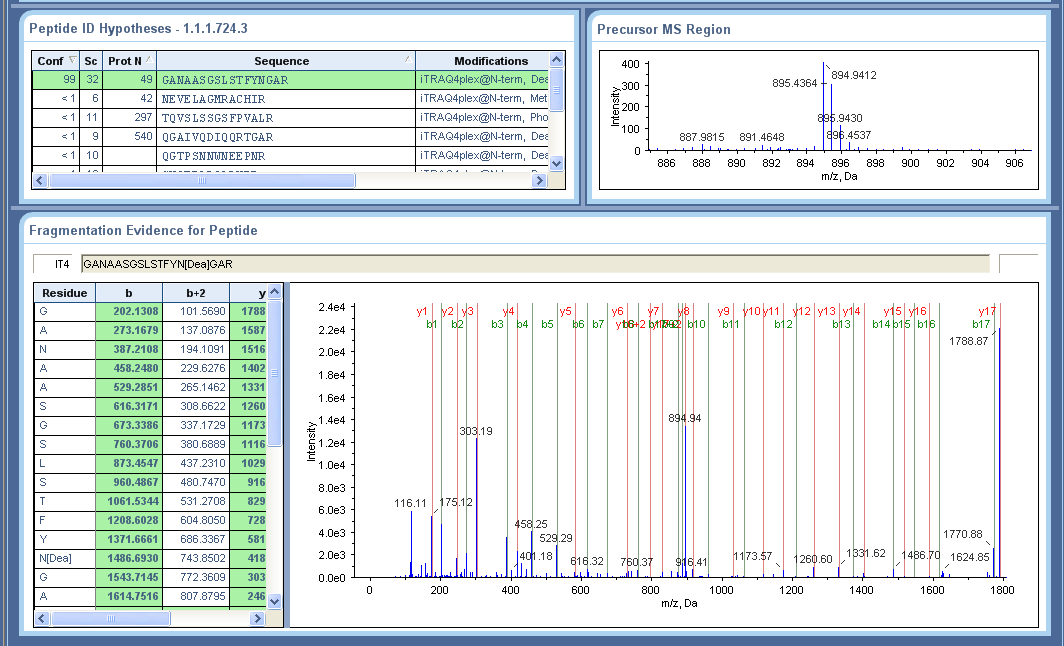
**

1. Afu2g09030| Aspergillus fumigatus secreted dipeptidyl peptidase (722 aa)

**
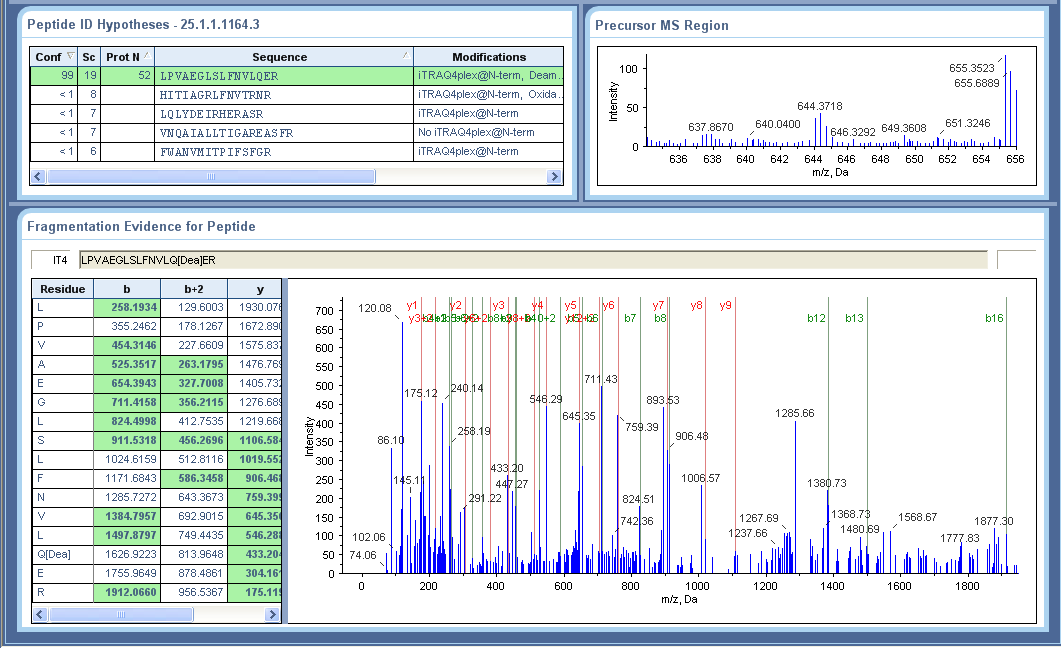
**

1. Afu4g03580| Aspergillus fumigatus alpha-galactosidase (892 aa)

IGN#DIIPAWR

**
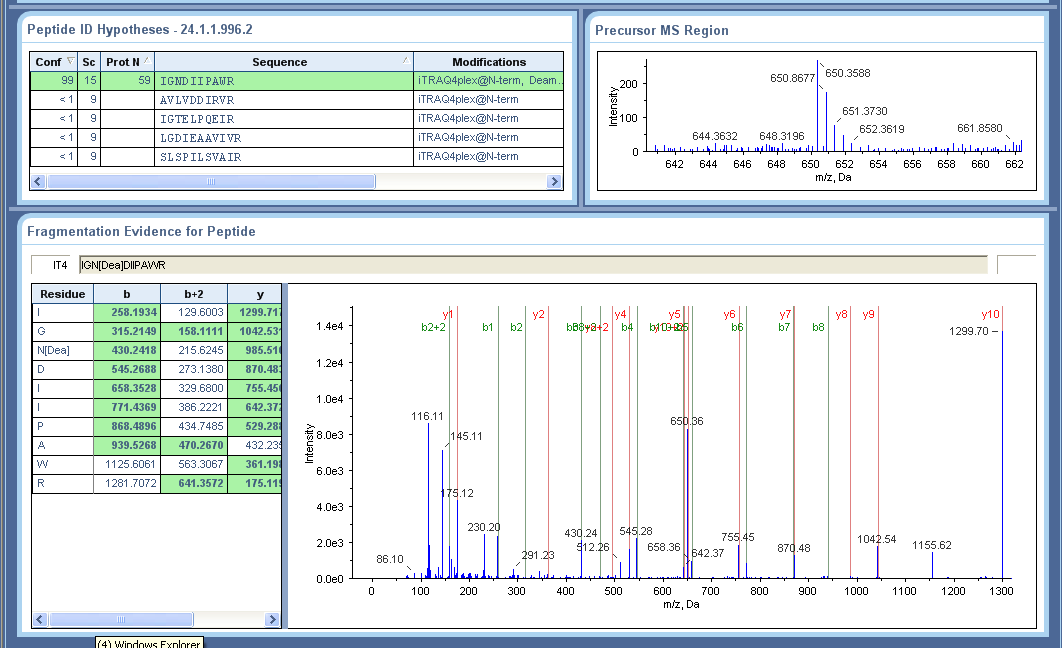
**

**
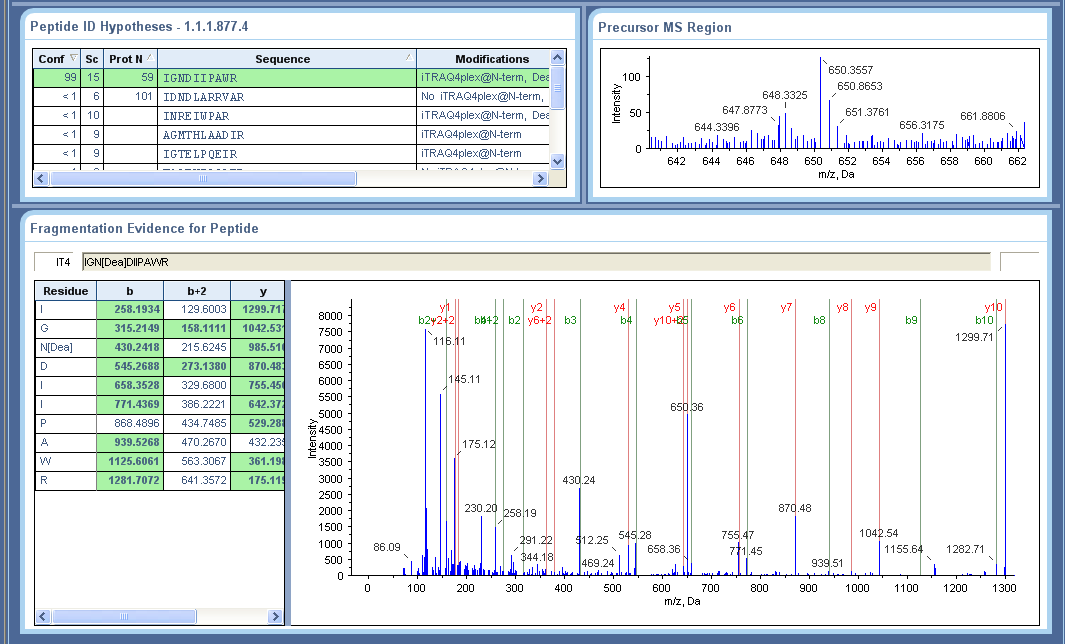
**

1. Afu6g03620| Aspergillus fumigatus isoamyl alcohol oxidase (572 aa)

**
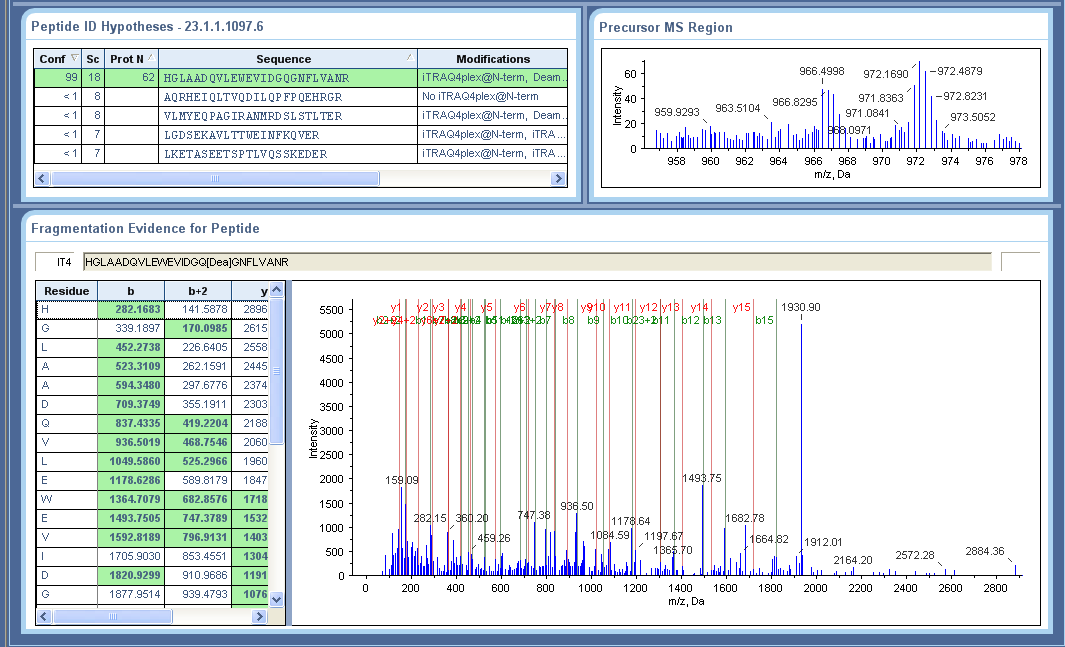
**

1. Afu6g03620| Aspergillus fumigatus isoamyl alcohol oxidase (572 aa)

SVVQTN#NAELTAAYR

**
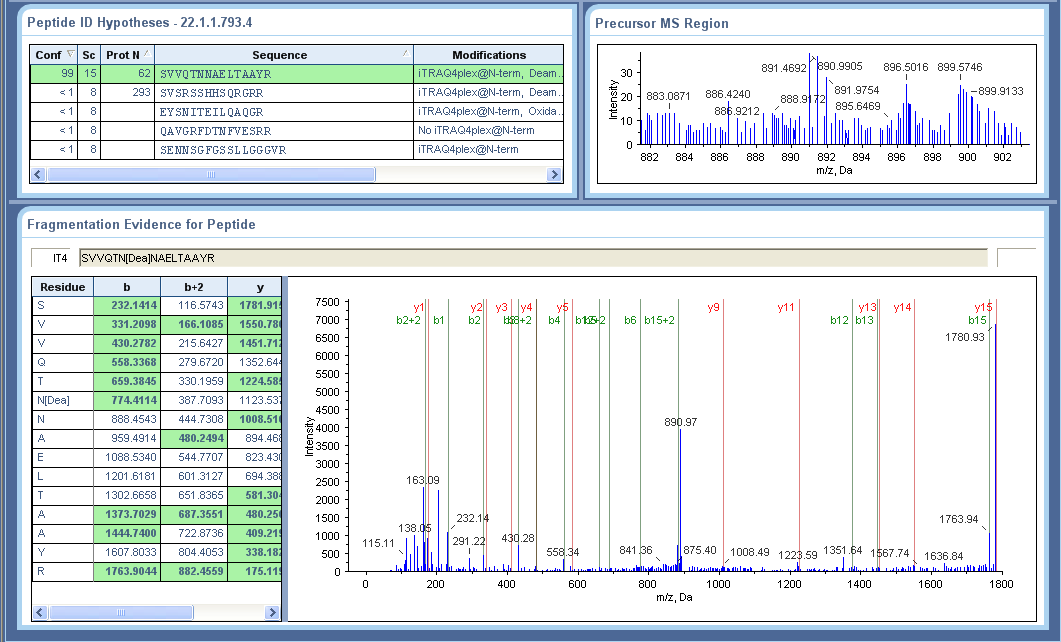
**

**
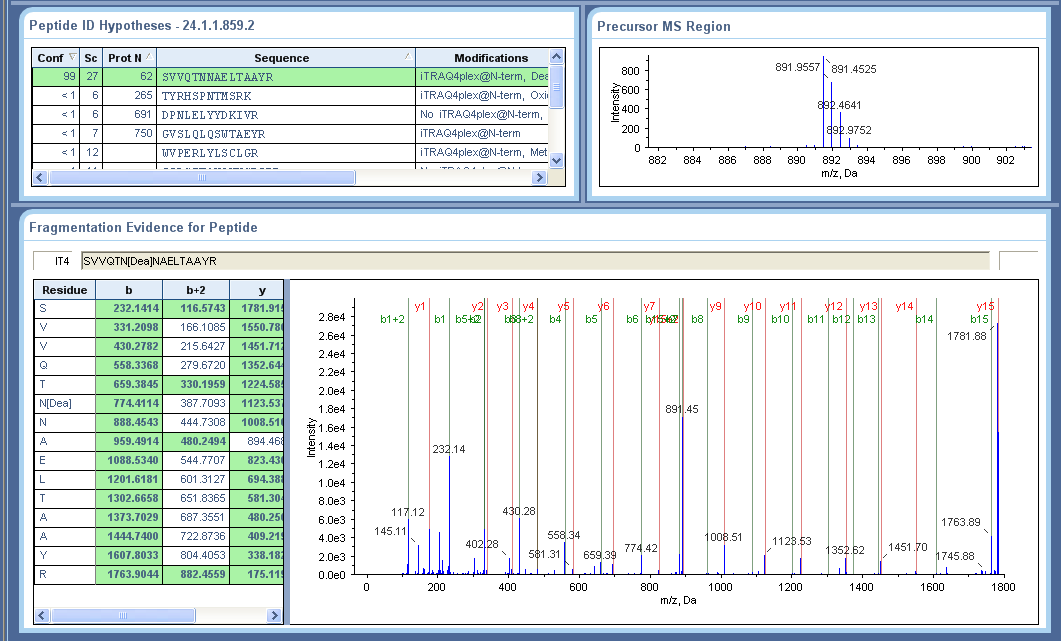
**

**
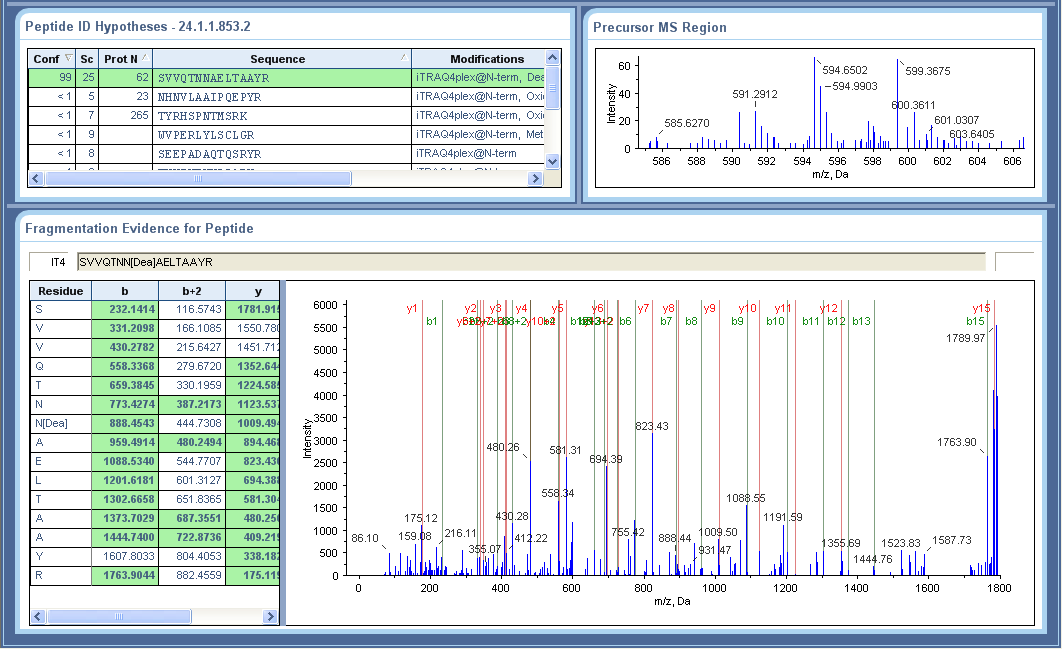
**

**
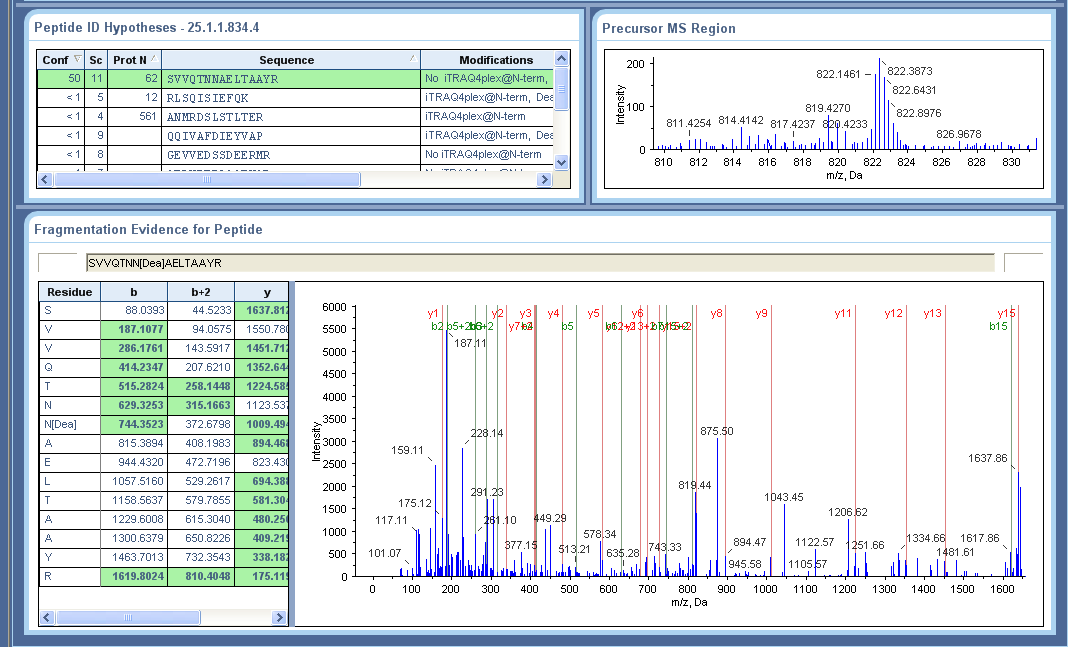
**

1. Afu2g00920| Aspergillus fumigatus glycosyl hydrolase, putative (397 aa)

SQTTLSNFPNGMGN#TVIALSDSNPNNLFEASNVYR

**
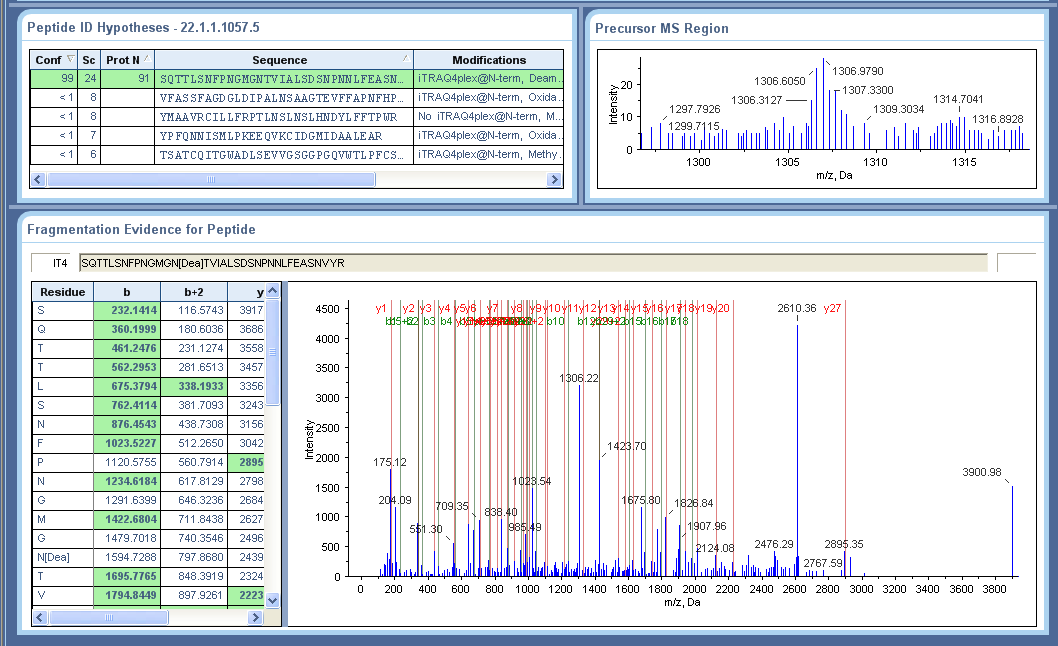
**

1. Afu2g14480| Aspergillus fumigatus oxidoreductase, FAD-binding, putative (474 aa)

GGANN#FGIVTNFIFR

**
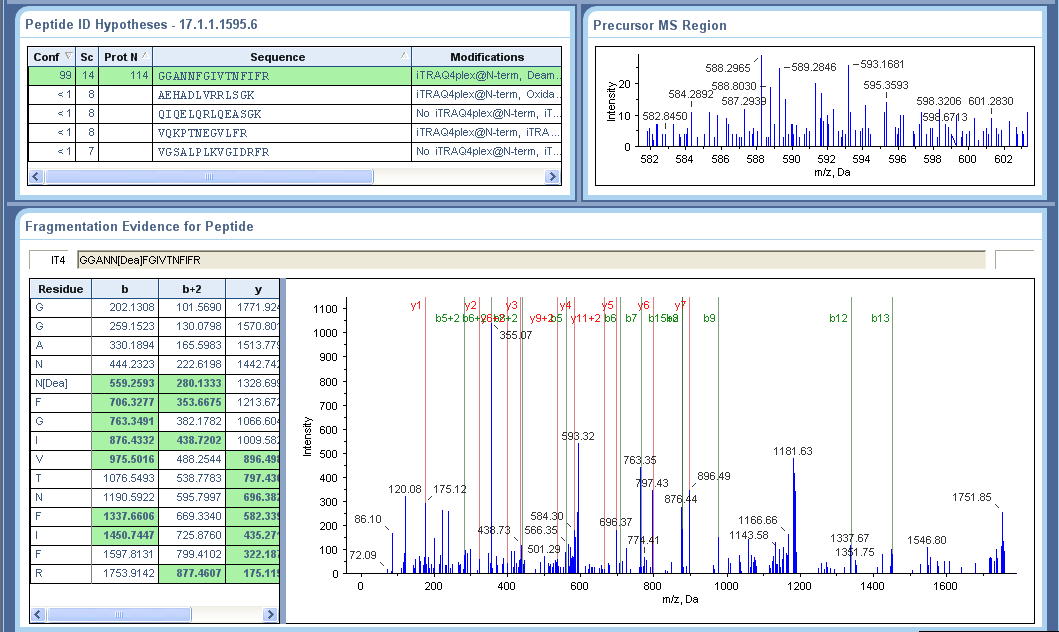
**

**
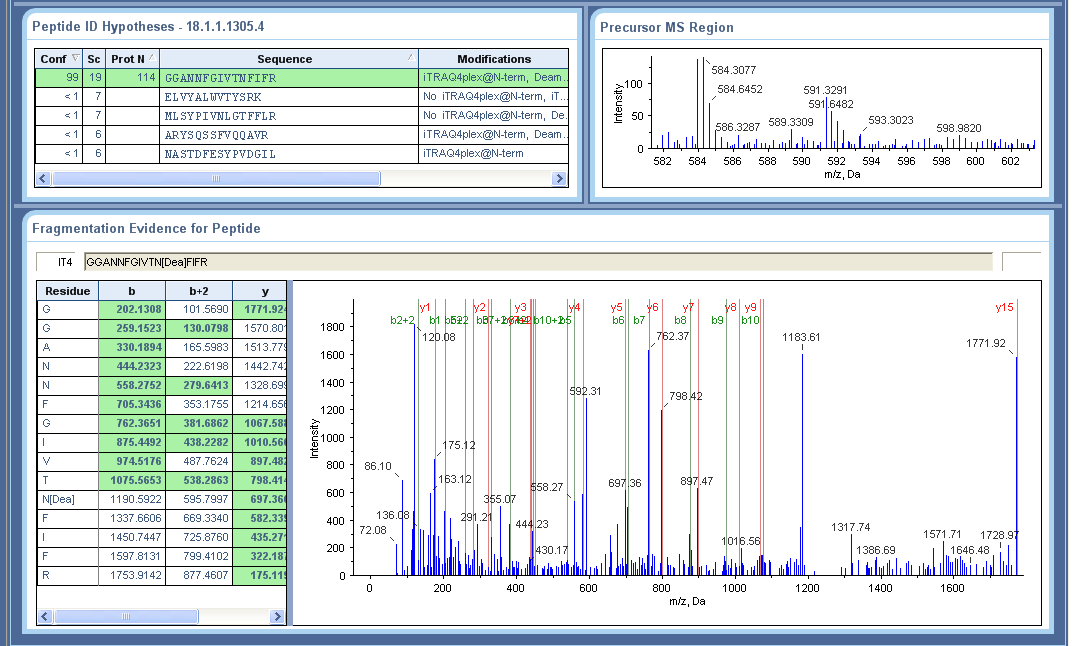
**

**
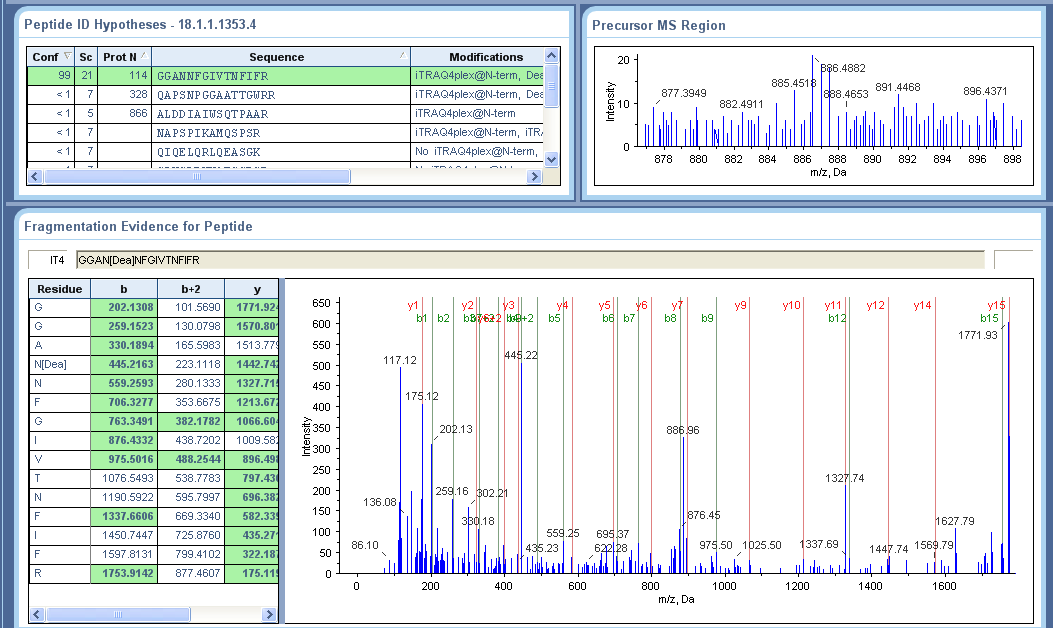
**

1. Afu1g05290| Aspergillus fumigatus endo-1,3(4)-beta-glucanase, putative (357 aa)

NTAQDTGLIFAN#GAVYMGVDHTNVAGSSGR

**
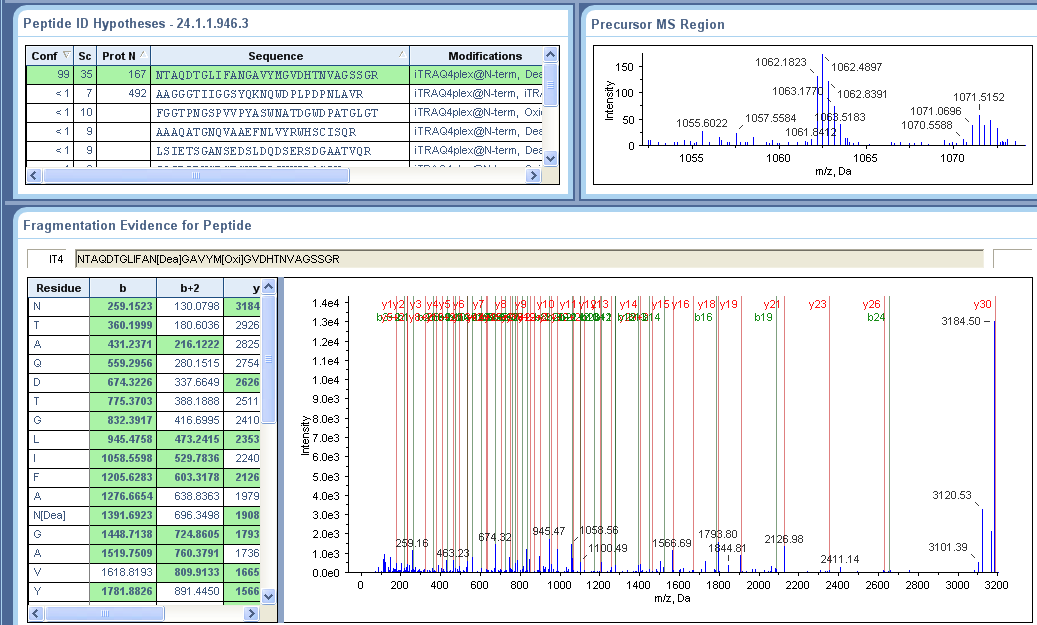
**

**
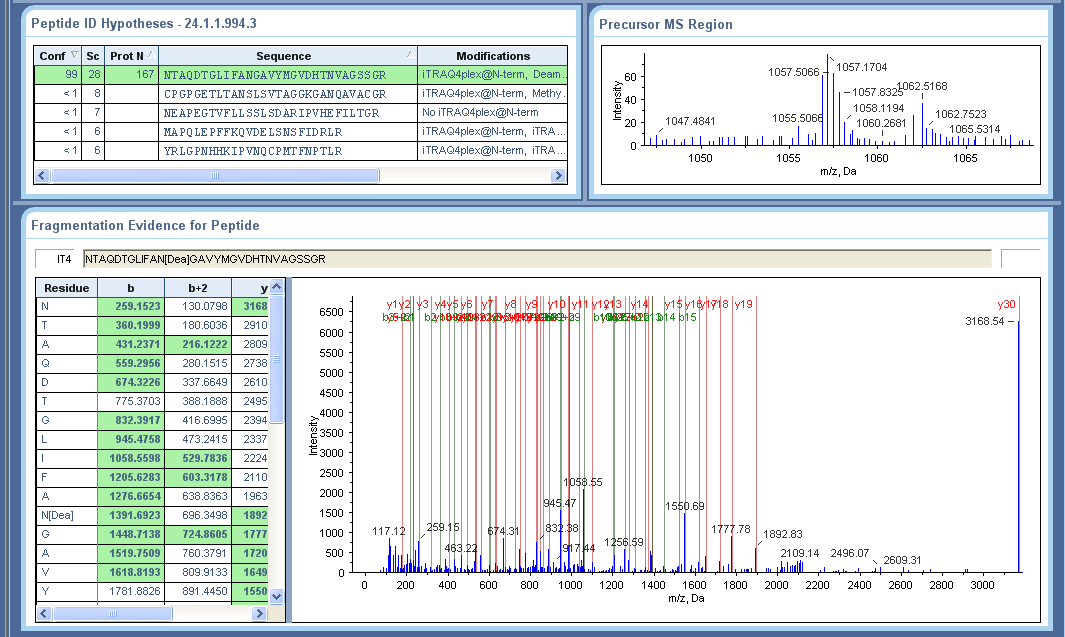
**

1. Afu3g00470| Aspergillus fumigatus endo-1,4-beta-xylanase, putative (314 aa)

GTVTSDGSTYDIYEHQQ#VNQPSIVGTATFN#QYWSIR

**
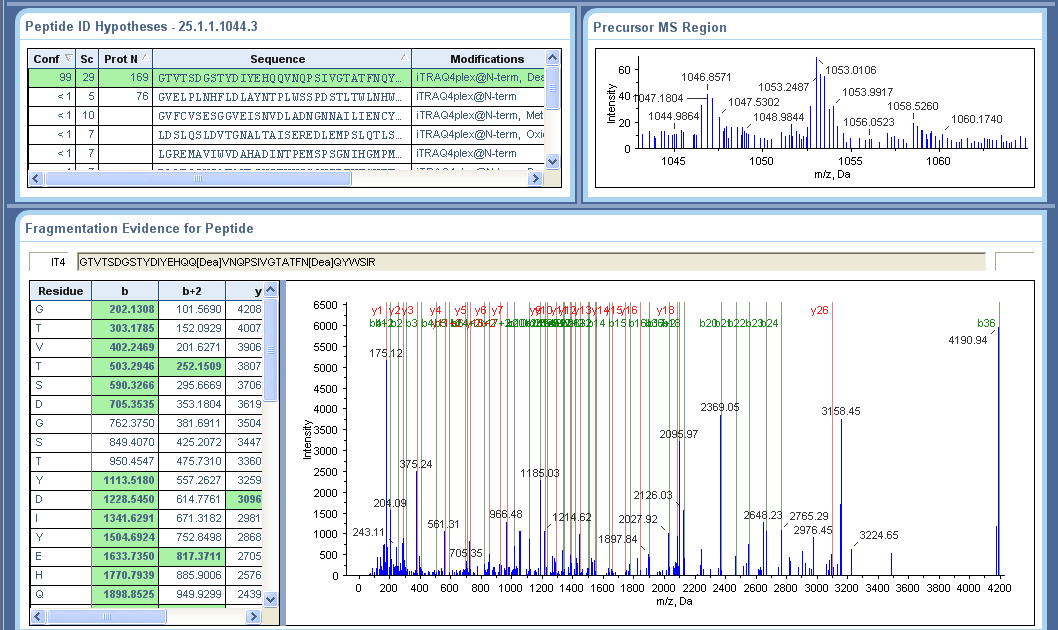
**

1. Afu1g15670| Aspergillus fumigatus laccase (597 aa)

**
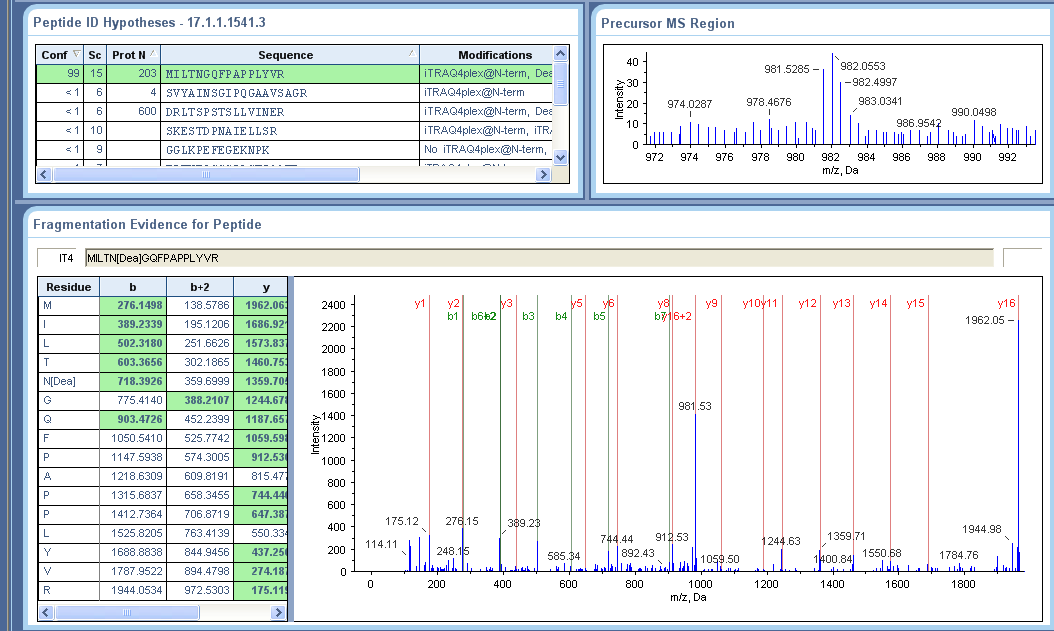
**

1. Afu6g07070| Aspergillus fumigatus cellobiohydrolase D (453 aa)

**
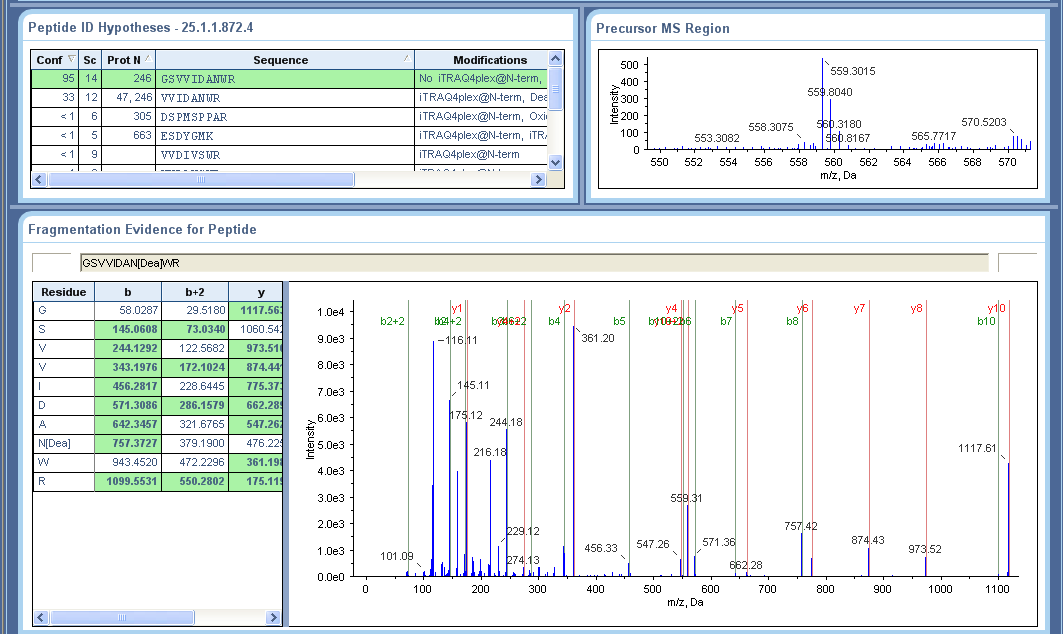
**
