# Supplementary material for: Data for iTRAQ secretomic analysis of Aspergillus fumigatus in response to different carbon sources
Source: Data Brief. 2015 Mar 11;3:175–9. doi: 10.1016/j.dib.2015.03.001 (PMC4510139; doi:10.1016/j.dib.2015.03.001)
Supplement: Supplementary file 1 — Supplementary Data [file mmc1.zip › TableS3.doc]

Table S3. iTRAQ quantified amylases, proteins involved in cell morphogenesis and hypothetical protein in different substrate conditions.

| **Unused** | **Total** | **%Cov** | **Accession No.** | **Name** | **Peptide**  **(95%)** | **Cellulose** | **Xylan** | **Starch** |
| --- | --- | --- | --- | --- | --- | --- | --- | --- |
| **Amylases** | | |  |  |  |  |  |  |
| 18.87±4.26 | 18.88±4.26 | 31.86±3.83 | Afu2g00710 | alpha-amylase, putative | 15±5 | 1.06±0.08 | 0.57±0.05 | 1.25±0.23 |
| 21.32±3.08 | 21.32±3.08 | 48.66±8.98 | Afu3g00900 | alpha-amylase AmyA | 17±4 | 0.76±0.04 | 1.10±0.16 | 1.32±0.21 |
| **Lipiases, Chitinases and phosphatases** | | | |  |  |  |  |  |
| 4.26±1.70 | 4.26±1.70 | 37.72±5.90 | Afu2g03110 | alkaline phosphatase | 2±1 | 1.06±0.24 | 5.89±0.79 | 1.11±0.21 |
| 7.51±2.61 | 7.51±2.61 | 45.18±12.80 | Afu1g17250 | conidial hydrophobin RodB | 4±1 | 0.52±0.09 | 0.27±0.04 | 0.31±0.05 |
| 5.20±0.88 | 5.21±0.88 | 31.61±5.88 | Afu8g00930 | chitosanase, putative | 3±1 | 0.38±0.01 | 0.35±0.06 | 0.50±0.04 |
| 8.66±3.66 | 8.66±3.66 | 36.87±6.02 | Afu5g02040 | extracellular lipase, putative | 14±6 | 0.72±0.11 | 4.10±1.80 | 0.60±0.07 |
| 7.22±1.40 | 7.22±1.40 | 29.41±5.98 | Afu1g03570 | acid phosphatase PHOa | 5±1 | 0.67±0.04 | 1.13±0.19 | 1.16±0.13 |
| 11.15±2.04 | 11.15±2.04 | 45.06±5.65 | Afu4g01290 | endo-chitosanase, pseudogene | 13±4 | 1.15±0.04 | 0.23±0.04 | 0.60±0.06 |
| 10.57±3.17 | 11.45±3.23 | 50.81±14.57 | Afu3g11280 | class V chitinase, putative | 9±4 | 1.13±0.19 | 2.61±0.73 | 3.19±0.46 |
| 7.97±1.50 | 7.97±1.50 | 44.88±8.22 | Afu1g16480 | acid phosphatase, putative | 4±1 | 1.09±0.08 | 1.56±0.98 | 1.65±0.20 |
| 14.15±1.92 | 14.15±1.92 | 43.12±4.98 | Afu3g07160 | class V chitinase, putative | 12±4 | 0.74±0.05 | 2.19±0.58 | 1.53±0.33 |
| 9.05±2.56 | 9.17±2.50 | 40.63±7.73 | Afu1g07440 | Hsp70 chaperone | 5±1 | 0.96±0.02 | 1.35±0.18 | 3.16±0.34 |
| 15.18±4.17 | 15.18±4.17 | 52.04±9.63 | Afu3g01530 | phosphatidylglycerol specific phospholipase C, | 20±6 | 1.28±0.06 | 1.84±0.30 | 2.47±0.46 |
| 17.55±3.60 | 17.55±3.60 | 47.34±3.18 | Afu4g03660 | acid phosphatase, putative | 25±7 | 0.81±0.03 | 0.58±0.09 | 1.63±0.24 |
| 39.47±5.47 | 39.47±5.47 | 72.40±1.89 | Afu8g01410 | class V chitinase ChiB1 | 83±25 | 1.78±0.46 | 2.63±0.62 | 2.64±0.57 |
| 29.09±3.50 | 29.09±3.50 | 80.11±11.16 | Afu6g07940 | lactoylglutathione lyase | 23±4 | 1.02±0.05 | 0.80±0.08 | 2.88±0.18 |
| 7.53±3.43 | 7.53±3.43 | 32.94±6.59 | Afu3g14680 | lysophospholipase Plb3 | 4±2 | 0.70±0.05 | 1.67±0.12 | 1.83±0.14 |
| 2.80±0.66 | 2.80±0.66 | 9.46±0.81 | Afu5g03760 | class III chitinase ChiA1 | 3±1 | 0.96±0.13 | 5.23±1.10 | 0.76±0.07 |
| **Hypothetical proteins** | | | |  |  |  |  |  |
| 3.19±1.17 | 3.19±1.17 | 15.03±5.07 | Afu8g02510 | hypothetical protein | 2±1 | 2.68±0.20 | 5.34±0.88 | 0.79±0.09 |
| 6.24±3.08 | 6.24±3.08 | 55.56±9.87 | Afu2g05720 | conserved hypothetical protein | 6±3 | 0.82±0.13 | 0.70±0.04 | 2.18±0.77 |
| 40.90±6.62 | 40.90±6.62 | 68.56±3.40 | Afu2g05240 | conserved hypothetical protein | 64±20 | 0.65±0.06 | 0.29±0.02 | 0.51±0.05 |
| 36.89±8.46 | 36.89±8.46 | 46.39±3.20 | Afu6g03150 | hypothetical protein | 44±15 | 0.98±0.05 | 5.66±1.07 | 0.78±0.06 |
| 24.40±4.32 | 24.40±4.32 | 81.67±5.49 | Afu3g01130 | hypothetical protein | 17±5 | 0.84±0.02 | 3.16±0.24 | 1.29±0.07 |
| 24.65±2.01 | 24.65±2.01 | 59.55±4.11 | Afu3g02600 | hypothetical protein | 35±9 | 0.49±0.05 | 1.59±0.14 | 0.38±0.04 |
| 20.79±5.77 | 20.79±5.77 | 48.58±7.94 | Afu7g04680 | conserved hypothetical protein | 11±3 | 1.14±0.04 | 3.65±0.58 | 0.80±0.08 |
| 13.75±5.95 | 13.75±5.95 | 45.61±7.20 | Afu5g01190 | conserved hypothetical protein | 8±3 | 1.84±0.35 | 3.98±1.60 | 0.61±0.07 |
| 15.01±0.96 | 15.01±0.96 | 64.34±9.28 | Afu5g01120 | conserved hypothetical protein | 10±1 | 0.55±0.05 | 0.43±0.03 | 0.67±0.06 |
| 13.52±2.90 | 13.52±2.90 | 32.18±8.18 | Afu3g07870 | conserved hypothetical protein | 9±2 | 1.47±0.16 | 2.28±0.34 | 0.93±0.08 |
| 11.88±2.27 | 11.88±2.27 | 42.38±10.45 | Afu5g10930 | hypothetical protein | 12±4 | 0.53±0.05 | 0.53±0.05 | 1.08±0.13 |
| 9.29±3.09 | 9.29±3.09 | 34.26±9.01 | Afu3g12690 | conserved hypothetical protein | 6±3 | 1.02±0.06 | 1.15±0.10 | 1.78±0.50 |
| 11.04±1.54 | 11.04±1.54 | 59.75±11.75 | Afu5g14560 | hypothetical protein | 7±2 | 1.41±0.05 | 2.15±0.27 | 1.06±0.09 |
| 9.30±2.48 | 9.30±2.48 | 32.11±4.55 | Afu6g06560 | conserved hypothetical protein | 6±1 | 0.97±0.08 | 1.95±0.45 | 0.81±0.11 |
| 11.36±4.17 | 11.36±4.17 | 26.77±4.14 | Afu4g13990 | hypothetical protein | 21±6 | 0.40±0.06 | 0.83±0.07 | 0.73±0.06 |
| 5.96±1.93 | 5.96±1.93 | 18.83±3.60 | Afu7g05340 | conserved hypothetical protein | 3±1 | 0.68±0.06 | 0.84±0.11 | 0.95±0.31 |
| 8.53±0.87 | 8.53±0.87 | 56.57±6.12 | Afu2g17630 | hypothetical protein | 7±2 | 0.92±0.06 | 4.88±0.91 | 0.87±0.08 |
| 6.51±1.65 | 6.51±1.65 | 46.59±4.10 | Afu5g01420 | conserved hypothetical protein | 4±1 | 1.02±0.33 | 0.75±0.13 | 0.91±0.13 |
| 5.80±1.81 | 5.80±1.81 | 47.76±11.07 | Afu5g03520 | hypothetical protein | 3±1 | 0.94±0.05 | 1.14±0.10 | 1.05±0.21 |
| 5.32±0.77 | 5.32±0.77 | 57.11±20.77 | Afu6g00180 | hypothetical protein | 16±6 | 0.66±0.04 | 1.21±0.13 | 1.82±0.18 |
| 9.03±1.92 | 9.03±1.92 | 31.32±3.36 | Afu5g02100 | hypothetical protein | 6±1 | 1.05±0.07 | 3.21±0.53 | 2.96±0.50 |
| 6.48±2.16 | 6.48±2.16 | 31.56±4.72 | Afu1g16430 | hypothetical protein | 4±1 | 1.34±0.24 | 1.42±0.42 | 2.32±0.57 |
| 6.09±1.44 | 6.09±1.44 | 57.80±13.78 | Afu8g00430 | hypothetical protein | 5±1 | 1.01±0.33 | 0.93±0.33 | 1.66±0.56 |
| 5.75±0.74 | 5.75±0.74 | 31.41±6.26 | Afu6g14470 | hypothetical protein | 3±0 | 1.71±0.17 | 7.23±2.00 | 1.18±0.16 |
| 5.00±1.00 | 5.00±1.00 | 27.40±3.51 | Afu1g09960 | conserved hypothetical protein | 4±2 | 1.21±0.37 | 0.85±0.06 | 2.50±0.28 |
| 7.38±2.20 | 6.07±1.62 | 18.82±9.49 | Afu3g03610 | hypothetical protein | 3±1 | 0.94±0.04 | 1.06±0.27 | 1.14±0.28 |
| 4.15±1.26 | 4.15±1.26 | 50.30±9.53 | Afu4g08450 | conserved hypothetical protein | 4±1 | 0.51±0.07 | 0.54±0.10 | 0.47±0.05 |
| 4.00±0.00 | 4.00±0.00 | 18.73±3.65 | Afu6g02010 | hypothetical protein | 4±0 | 0.98±0.13 | 5.24±0.60 | 1.39±0.11 |
| 4.04±0.04 | 4.04±0.04 | 35.44±3.10 | Afu3g00960 | conserved hypothetical protein | 3±1 | 0.96±0.09 | 2.07±0.34 | 2.30±0.45 |
| 4.04±1.39 | 4.04±1.39 | 57.18±12.84 | Afu4g08310 | conserved hypothetical protein | 3±1 | 0.66±0.05 | 0.74±0.07 | 1.32±0.18 |
| 3.74±1.99 | 3.74±1.99 | 23.28±13.73 | Afu3g10390 | conserved hypothetical protein | 2±1 | 1.13±0.22 | 1.05±0.15 | 1.58±0.55 |
| 5.00±1.73 | 5.00±1.73 | 18.99±3.91 | Afu6g14400 | conserved hypothetical protein | 3±1 | 1.47±0.01 | 1.25±0.12 | 0.87±0.10 |
| 3.44±0.60 | 3.44±0.60 | 39.15±6.72 | Afu1g02290 | conserved hypothetical protein | 4±2 | 0.98±0.10 | 1.19±0.23 | 1.52±0.26 |
| 5.00±1.00 | 5.00±1.00 | 62.05±11.48 | Afu3g14940 | hypothetical protein | 3±1 | 0.94±0.14 | 1.05±0.10 | 0.90±0.09 |
| 9.28±3.30 | 9.28±3.30 | 50.87±12.89 | Afu2g15770 | conserved hypothetical protein | 5±2 | 1.07±0.12 | 1.90±0.38 | 2.48±0.56 |
| 3.62±0.56 | 3.62±0.56 | 24.72±3.98 | Afu2g15420 | hypothetical protein | 3±1 | 1.20±0.12 | 1.03±0.23 | 0.56±0.08 |
| 6.24±2.33 | 6.24±2.33 | 50.36±2.57 | Afu4g02840 | conserved hypothetical protein | 3±2 | 1.02±0.15 | 0.71±0.14 | 1.57±0.31 |
| 8.50±34.70 | 3.67±0.85 | 12.16±5.44 | Afu8g00630 | conserved hypothetical protein, putative | 2±1 | 1.57±0.64 | 4.12±2.52 | 3.07±2.72 |
| 3.10±1.02 | 3.10±1.02 | 32.34±8.54 | Afu2g15710 | hypothetical protein | 2±1 | 1.02±0.10 | 1.79±0.50 | 1.91±0.64 |
| 2.76±0.44 | 2.00±0.00 | 11.30±13.15 | Afu2g09140 | conserved hypothetical protein | 2±0 | 1.02±0.00 | 1.65±0.00 | 1.63±0.00 |
| 2.06±0.05 | 2.10±0.09 | 29.09±8.16 | Afu2g01840 | conserved hypothetical protein | 2±0 | 1.62±0.50 | 1.40±0.36 | 3.10±0.87 |
| 3.50±0.87 | 3.50±0.87 | 28.33±2.92 | Afu6g11720 | hypothetical protein | 2±0 | 1.03±0.25 | 1.20±0.15 | 1.37±0.21 |
| 2.12±0.41 | 2.12±0.41 | 14.00±6.15 | Afu5g14420 | hypothetical protein | 2±0 | 0.49±0.08 | 0.65±0.08 | 1.51±0.13 |
| 3.41±1.41 | 3.41±1.41 | 20.87±5.92 | Afu1g05270 | conserved hypothetical protein | 4±1 | 1.05±0.09 | 2.46±0.31 | 1.17±0.13 |
| 2.00±0.00 | 2.00±0.00 | 31.39±12.63 | Afu6g10840 | hypothetical protein | 2±0 | 0.88±0.09 | 1.90±0.33 | 5.26±1.22 |
| 2.00±0.00 | 2.00±0.00 | 22.38±14.72 | Afu2g12000 | conserved hypothetical protein | 2±1 | 0.51±0.01 | 0.31±0.04 | 0.55±0.07 |
