# Supplementary material for: Data for iTRAQ secretomic analysis of Aspergillus fumigatus in response to different carbon sources
Source: Data Brief. 2015 Mar 11;3:175–9. doi: 10.1016/j.dib.2015.03.001 (PMC4510139; doi:10.1016/j.dib.2015.03.001)
Supplement: Supplementary file 1 — Supplementary Data [file mmc1.zip › TableS2.doc]

Table S2. Lignin degrading enzymes, peptidases and proteases by *A. fumigatus* LF9 when different substrates were used as a main carbon sources.

| **Unused** | **Total** | **%Cov** | **Accession No.** | **Name** | **Peptide**  **(95%)** | **Cellulose** | **Xylan** | **Starch** |
| --- | --- | --- | --- | --- | --- | --- | --- | --- |
| **Lignin degrading enzymes** | | |  |  |  |  |  |  |
| 13.95±3.54 | 13.95±3.54 | 32.25±5.81 | Afu1g01180 | isoamyl alcohol oxidase | 9±3 | 1.17±0.08 | 1.24±0.23 | 1.45±0.21 |
| 20.69±4.39 | 20.69±4.39 | 50.48±7.37 | Afu6g03620 | isoamyl alcohol oxidase | 31±10 | 0.80±0.01 | 1.09±0.10 | 1.29±0.11 |
| 42.64±5.93 | 42.64±5.93 | 73.38±7.29 | Afu8g01670 | bifunctional catalase-peroxidase Cat2 | 34±7 | 1.15±0.06 | 1.21±0.10 | 3.00±0.42 |
| 15.02±2.71 | 15.02±2.71 | 89.08±8.53 | Afu5g09240 | Cu,Zn superoxide dismutase | 14±4 | 0.86±0.04 | 4.08±0.42 | 0.74±0.18 |
| 14.22±2.29 | 14.22±2.29 | 53.17±12.29 | Afu2g14480 | oxidoreductase, FAD-binding, putative | 14±4 | 0.95±0.01 | 1.03±0.16 | 0.91±0.10 |
| 11.79±0.84 | 11.79±0.84 | 72.50±4.60 | Afu1g14550 | Mn superoxide dismutase MnSOD | 9±2 | 1.08±0.10 | 0.75±0.08 | 2.17±0.24 |
| 10.00±0.73 | 10.00±0.73 | 57.17±8.98 | Afu4g09110 | cytochrome c peroxidase, putative | 7±1 | 1.03±0.02 | 4.38±0.63 | 2.56±0.37 |
| 29.63±3.42 | 29.63±3.42 | 65.29±10.53 | Afu3g00840 | FAD-dependent oxygenase, putative | 44±10 | 0.63±0.05 | 0.97±0.13 | 0.81±0.10 |
| 11.10±2.09 | 11.10±2.09 | 60.37±4.97 | Afu4g11580 | Mn superoxide dismutase | 6±1 | 1.02±0.05 | 0.89±0.17 | 2.91±0.91 |
| 3.77±1.77 | 3.77±1.77 | 29.57±8.05 | Afu1g15670 | laccase | 2±1 | 0.43±0.08 | 0.52±0.04 | 0.33±0.02 |
| 233.67±64.97 | 2.87±1.61 | 10.17±11.20 | Afu7g05070 | FAD dependent oxidoreductase, putative | 2±0 | 1.09±0.00 | 2.67±1.52 | 7.27±5.87 |
| 10.53±4.52 | 10.53±4.52 | 39.87±8.66 | Afu3g02270 | mycelial catalase Cat1 | 5±2 | 1.83±0.05 | 2.83±0.39 | 0.74±0.11 |
| 2.01±0.00 | 227.51±25.50 | 18.48±6.47 | Afu4g00610 | aryl-alcohol dehydrogenase, putative | 2±0 | 1.04±0.00 | 0.82±0.00 | 1.42±0.00 |
| 243.25±76.43 | 2.27±0.34 | 16.18±20.02 | Afu1g15960 | glutathione reductase | 3±0 | 0.75±0.00 | 0.55±0.00 | 0.88±0.00 |
| 3.14±0.90 | 3.14±0.90 | 37.64±8.99 | Afu1g15610 | oxidoreductase, | 2±1 | 1.54±0.12 | 1.00±0.19 | 1.05±0.19 |
| 2.98±0.73 | 2.98±1.73 | 34.70±17.35 | Afu7g01010 | alcohol dehydrogenase, putative | 2±1 | 1.29±0.10 | 1.10±0.04 | 1.14±0.02 |
| 2.02±0.02 | 2.02±0.02 | 20.05±4.31 | Afu2g04710 | cytochrome b5, putative | 2±0 | 2.13±0.64 | 4.18±1.44 | 1.06±0.29 |
| **Peptidases and Proteases** | | |  |  |  |  |  |  |
| 58.93±12.65 | 58.93±12.65 | 62.59±8.26 | Afu4g09030 | aminopeptidase | 48±14 | 1.01±0.04 | 1.47±0.16 | 3.31±0.31 |
| 36.78±11.98 | 36.78±11.98 | 60.05±5.90 | Afu7g05930 | metallopeptidase MepB | 23±9 | 1.03±0.06 | 1.44±0.18 | 2.64±0.19 |
| 30.41±6.90 | 30.41±6.90 | 54.61±7.48 | Afu4g06140 | dipeptidyl peptidase III | 22±6 | 1.05±0.06 | 1.35±0.15 | 2.27±0.25 |
| 29.95±5.32 | 29.95±5.32 | 71.09±4.54 | Afu4g11800 | alkaline serine protease Alp1 | 92±36 | 0.75±0.02 | 2.21±0.37 | 1.28±0.21 |
| 25.83±6.31 | 25.83±6.31 | 59.86±8.32 | Afu5g08050 | aminopeptidase P, putative | 16±7 | 0.97±0.06 | 1.70±0.17 | 2.08±0.33 |
| 20.45±3.67 | 20.45±3.67 | 44.43±5.79 | Afu5g03990 | aspartyl aminopeptidase | 14±4 | 1.63±0.32 | 2.07±0.21 | 1.74±0.32 |
| 26.25±6.38 | 26.26±6.38 | 50.00±4.73 | Afu5g04330 | aminopeptidase B | 15±4 | 0.96±0.04 | 1.74±0.18 | 2.42±0.21 |
| 18.67±4.24 | 18.67±4.24 | 45.51±5.96 | Afu5g13300 | aspartic endopeptidase Pep1 | 12±4 | 1.13±0.06 | 0.52±0.03 | 0.64±0.05 |
| 21.43±5.53 | 21.43±5.53 | 42.09±6.52 | Afu2g09030 | secreted dipeptidyl peptidase | 15±5 | 1.49±0.11 | 2.14±0.33 | 2.25±0.48 |
| 12.78±2.27 | 12.78±2.27 | 34.99±8.53 | Afu5g01200 | carboxypeptidase S1, putative | 9±3 | 0.67±0.05 | 1.12±0.09 | 0.59±0.06 |
| 11.21±2.27 | 11.21±2.27 | 37.50±4.89 | Afu4g03490 | tripeptidyl-peptidase | 7±2 | 0.32±0.00 | 0.09±0.01 | 0.09±0.01 |
| 19.49±1.57 | 19.49±1.57 | 38.71±2.39 | Afu3g07030 | glutaminase A | 15±3 | 2.18±0.08 | 2.93±0.26 | 0.73±0.06 |
| 14.58±3.91 | 14.58±3.91 | 68.81±7.17 | Afu3g08380 | inorganic diphosphatase, putative | 9±3 | 1.40±0.23 | 0.99±0.18 | 2.57±0.79 |
| 13.03±3.69 | 13.03±3.69 | 50.56±5.14 | Afu7g04760 | gamma-glutamyltranspeptidase | 8±3 | 0.97±0.04 | 1.95±0.18 | 1.30±0.17 |
| 8.28±2.53 | 8.28±2.53 | 43.38±8.56 | Afu3g08930 | tripeptidyl peptidase A | 4±1 | 0.62±0.06 | 0.61±0.02 | 0.57±0.03 |
| 11.75±2.83 | 11.75±2.83 | 40.30±7.49 | Afu8g07080 | elastinolytic metalloproteinase Mep | 10±4 | 0.93±0.32 | 1.62±0.41 | 0.81±0.05 |
| 5.04±1.76 | 5.04±1.76 | 35.10±8.17 | Afu2g01250 | serine peptidase, family S28, putative | 4±2 | 0.83±0.08 | 2.04±0.50 | 1.10±0.20 |
| 93.84±5.98 | 4.99±0.70 | 25.84±15.28 | Afu6g06800 | peptidase, putative | 3±1 | 1.49±0.00 | 0.70±0.09 | 0.64±0.05 |
| 4.65±0.95 | 4.65±0.95 | 29.32±3.40 | Afu6g00310 | serine carboxypeptidase | 3±1 | 1.15±0.09 | 0.51±0.05 | 0.38±0.09 |
| 3.43±0.80 | 3.43±0.80 | 23.88±5.62 | Afu3g02970 | aspergillopepsin, putative | 5±2 | 5.89±0.06 | 0.27±0.03 | 0.23±0.01 |
| 7.09±4.24 | 7.09±4.24 | 36.11±8.78 | Afu3g08290 | aspartyl aminopeptidase | 5±3 | 1.37±0.13 | 2.94±1.78 | 0.82±0.22 |
| 4.70±1.65 | 4.70±1.65 | 25.79±3.82 | Afu8g04730 | oligopeptidase family protein | 2±1 | 0.82±0.24 | 4.83±1.71 | 1.07±0.38 |
| 2.19±1.70 | 2.38±0.27 | 10.82±12.06 | Afu5g09210 | alkaline serine protease Alp2 | 2±0 | 1.44±0.00 | 1.44±0.00 | 0.93±0.00 |
| 2.02±0.00 | 2.21±1.99 | 3.79±1.76 | Afu4g14000 | tripeptidyl peptidase A | 2±0 | 1.29±0.00 | 0.79±0.00 | 0.66±0.00 |
| 2.70±1.65 | 4.06±1.67 | 24.29±12.92 | Afu6g10250 | alkaline serine protease AorO, putative | 2±1 | 1.09±0.03 | 1.68±0.58 | 1.18±0.66 |
| 2.00±0.00 | 2.00±0.00 | 20.90±3.92 | Afu8g04120 | carboxypeptidase S1, putative | 2±1 | 4.51±0.29 | 1.74±0.18 | 0.49±0.04 |
