# Supplementary material for: Data for iTRAQ secretomic analysis of Aspergillus fumigatus in response to different carbon sources
Source: Data Brief. 2015 Mar 11;3:175–9. doi: 10.1016/j.dib.2015.03.001 (PMC4510139; doi:10.1016/j.dib.2015.03.001)
Supplement: Supplementary file 1 — Supplementary Data [file mmc1.zip › TableS1.doc]

Table S1.iTRAQ quantified hemicellulases from *A. fumigatus* LF9 when different substrates were used as a main carbon sources.

| Unused | Total | %Cov | Accession No | Name | Family | Peptide (95%) | Cellulose | Xylan | Starch |
| --- | --- | --- | --- | --- | --- | --- | --- | --- | --- |
| 81.23±14.68 | 81.23±14.68 | 85.24±6.27 | Afu1g14560 | alpha-mannosidase | GH47 | 170±49 | 0.67±0.02 | 0.69±0.06 | 1.56±0.15 |
| 68.00±12.26 | 68.00±12.26 | 80.81±5.88 | Afu3g00320 | endo-1,4-beta-xylanase | GH11 | 135±44 | 1.96±0.17 | 6.90±0.51 | 0.66±0.05 |
| 44.21±8.10 | 44.21±8.10 | 67.13±5.85 | Afu6g13610 | endo-1,4-beta-xylanase, putative | GH10 | 84±28 | 0.75±0.05 | 7.07±1.30 | 0.78±0.08 |
| 38.48±5.65 | 38.48±5.65 | 70.20±6.46 | Afu3g02090 | beta-xylosidase | GH3 | 39±11 | 1.03±0.08 | 5.03±0.25 | 0.80±0.06 |
| 30.92±7.41 | 30.92±7.41 | 78.16±8.06 | Afu4g09480 | extracellular endo-1,4-beta-xylanase, putative | GH10 | 29±10 | 1.77±0.59 | 7.58±1.24 | 0.87±0.10 |
| 28.76±7.42 | 28.76±7.42 | 48.12±8.38 | Afu1g10790 | alpha-1,2-mannosidase family protein, putative | GH92 | 19±7 | 0.69±0.03 | 0.72±0.05 | 1.00±0.05 |
| 25.71±5.00 | 25.71±5.00 | 49.95±5.47 | Afu6g14620 | alpha-L-arabinofuranosidase | NA | 28±10 | 2.59±0.25 | 6.18±0.54 | 0.71±0.09 |
| 24.93±7.90 | 24.93±7.90 | 62.80±8.13 | Afu6g00770 | extracellular arabinanase, putative | GH43 | 41±16 | 0.72±0.07 | 0.53±0.09 | 0.86±0.16 |
| 20.32±2.51 | 20.32±2.51 | 40.24±8.27 | Afu4g03580 | alpha-galactosidase | NA | 20±5 | 0.93±0.08 | 6.17±0.72 | 0.77±0.07 |
| 19.27±4.90 | 19.27±4.90 | 51.25±5.68 | Afu5g10520 | alpha-1,2-mannosidase family protein | GH92 | 12±4 | 0.99±0.08 | 1.24±0.13 | 1.90±0.28 |
| 18.81±4.50 | 18.81±4.50 | 35.45±6.98 | Afu1g14170 | beta-galactosidase, putative | GH35 | 12±3 | 1.32±0.03 | 2.17±0.43 | 1.00±0.13 |
| 18.58±4.39 | 18.58±4.39 | 53.82±11.16 | Afu8g04710 | xylosidase | GH43 | 14±4 | 0.85±0.04 | 6.33±0.90 | 0.86±0.07 |
| 18.41±1.71 | 18.41±1.71 | 68.05±6.85 | Afu4g03240 | cell wall galactomannoprotein Mp1 | NA | 22±5 | 1.22±0.18 | 5.02±1.14 | 1.39±0.21 |
| 15.10±2.38 | 15.11±2.38 | 51.56±8.20 | Afu6g02560 | alpha-galactosidase | NA | 8±1 | 1.11±0.11 | 1.92±0.32 | 1.68±0.33 |
| 12.72±2.29 | 12.72±2.29 | 63.91±11.09 | Afu2g15430 | L-xylulose reductase | NA | 15±4 | 1.01±0.07 | 0.73±0.10 | 2.07±0.47 |
| 12.31±2.21 | 12.31±2.21 | 36.62±5.69 | Afu6g13760 | alpha-1,2-mannosidase, putative subfamily | GH92 | 8±2 | 1.37±0.37 | 2.42±0.57 | 1.89±0.42 |
| 11.38±1.91 | 11.38±1.91 | 37.44±5.95 | Afu5g02130 | alpha-galactosidase | NA | 8±3 | 0.54±0.02 | 0.28±0.03 | 0.43±0.05 |
| 11.18±4.43 | 11.18±4.43 | 51.75±11.91 | Afu5g09860 | esterase, putative | NA | 6±3 | 1.13±0.09 | 1.06±0.24 | 1.55±0.51 |
| 10.40±2.90 | 10.40±2.90 | 40.15±7.09 | Afu3g00420 | acetyl xylan esterase | NA | 8±2 | 1.51±0.22 | 7.06±2.01 | 0.91±0.14 |
| 10.02±3.16 | 10.42±3.54 | 44.43±9.34 | Afu2g12770 | arabinofuranosidase | GH62 | 6±2 | 1.08±0.10 | 3.36±0.18 | 0.81±0.06 |
| 9.18±2.66 | 11.33±2.71 | 36.74±5.92 | Afu3g00470 | endo-1,4-beta-xylanase, putative | GH11 | 10±4 | 1.51±0.20 | 4.49±0.98 | 0.81±0.14 |
| 8.77±3.15 | 8.77±3.15 | 36.06±9.26 | Afu1g06910 | arabinogalactan endo-1,4-beta-galactosidase, | GH53 | 5±2 | 0.50±0.08 | 0.91±0.06 | 0.88±0.10 |
| 8.70±3.25 | 8.70±3.25 | 29.35±3.63 | Afu1g09900 | alpha-L-arabinofuranosidase | NA | 5±2 | 1.04±0.02 | 4.24±0.98 | 0.76±0.06 |
| 7.66±1.86 | 7.66±1.86 | 36.59±10.54 | Afu2g15160 | alpha-L-arabinofuranosidase A | NA | 5±1 | 1.00±0.08 | 4.09±0.80 | 0.86±0.07 |
| 7.00±3.31 | 7.00±3.31 | 58.48±12.31 | Afu3g14510 | rhamnogalacturonan acetylesterase, putative | NA | 4±2 | 0.51±0.06 | 0.26±0.02 | 0.56±0.11 |
| 6.60±2.24 | 6.60±2.24 | 27.08±2.94 | Afu6g08840 | beta-mannosidase | GH2 | 4±2 | 1.12±0.16 | 2.07±0.20 | 2.16±0.31 |
| 5.98±2.68 | 5.98±2.68 | 20.23±4.42 | Afu2g04480 | xylosidase/arabinosidase, putative | GH43 | 5±2 | 0.99±0.08 | 6.28±1.19 | 1.77±0.37 |
| 5.02±1.01 | 5.02±1.01 | 53.33±16.36 | Afu1g04820 | D-xylose reductase | NA | 3±1 | 1.07±0.09 | 3.49±0.85 | 1.01±0.03 |
| 4.23±1.41 | 4.23±1.41 | 24.14±8.65 | Afu2g00930 | Possible beta-xylosidase, family 43 of glycosyl | GH43 | 2±1 | 0.82±0.11 | 2.14±0.62 | 0.54±0.08 |
| 3.88±1.33 | 3.88±1.33 | 28.47±5.03 | Afu2g00650 | arabinosidase | GH43,GH32,GH62,GH68 | 2±1 | 0.76±0.05 | 1.91±0.42 | 1.49±0.18 |
| 3.51±1.66 | 3.51±1.66 | 27.58±6.78 | Afu1g17220 | extracellular polygalacturonase, putative | GH28 | 2±1 | 8.53±2.94 | 0.36±0.04 | 0.29±0.05 |
| 3.51±1.67 | 3.51±1.67 | 28.23±10.04 | Afu8g01970 | extracellular endo-polygalacturonase, putative | GH28 | 2±1 | 0.83±0.01 | 0.44±0.02 | 0.72±0.02 |
| 2.63±1.51 | 2.63±1.51 | 27.38±6.56 | Afu3g09070 | carboxylesterase, putative | NA | 2±1 | 1.08±0.18 | 2.69±0.90 | 0.68±0.05 |

NA: data not available

PL: Pectase lyase
