# Supplementary material for: Data for iTRAQ secretomic analysis of Aspergillus fumigatus in response to different carbon sources
Source: Data Brief. 2015 Mar 11;3:175–9. doi: 10.1016/j.dib.2015.03.001 (PMC4510139; doi:10.1016/j.dib.2015.03.001)
Supplement: Supplementary file 1 — Supplementary Data [file mmc1.zip › Table S4, Proteins from zymogram band.doc]

Table S4. The band showing cellulolytic activity was cut, digested and analyzed by LC-MS/MS. The proteins identified in the band are listed below along with their mass, protein score, emPAI and Peptides detected.

| Accession | GS | Name | Pro_Mass | Prot_score | emPAI | Peptides |
| --- | --- | --- | --- | --- | --- | --- |
| Afu3g00320 | Afu3g00320 | endo-1,4-beta-xylanase (XlnA), putative (229 aa) | 24536 | 1111 | 2.16 | 42 |
| Afu7g06150 | Afu7g06150 | endoglucanase, putative (235 aa) | 25883 | 742 | 2.37 | 33 |
| Afu1g16190 | Afu1g16190 | cell wall glucanase Crf1 (396 aa) | 40487 | 132 | 0.27 | 9 |
| Afu3g02970 | Afu3g02970 | aspergillopepsin, putative (270 aa) | 28225 | 52 | 0.25 | 3 |
| Afu1g07300 | Afu1g07300 | tetracycline transporter-like protein, putative (461 aa) | 49371 | 45 | 0.07 | 1 |
| Afu1g09060 | Afu1g09060 | conserved hypothetical protein (513 aa) | 57133 | 43 | 0.06 | 2 |
| Afu2g03620 | Afu2g03620 | oxidoreductase, short-chain | 53366 | 42 | 0.06 | 2 |
| Afu3g12510 | Afu3g12510 | vesicular fusion ATPase, putative (771 aa) | 84710 | 41 | 0.04 | 4 |
| Afu4g02960 | Afu4g02960 | UDP-sugar hydrolase, putative (397 aa) | 43996 | 39 | 0.08 | 1 |
| Afu3g09300 | Afu3g09300 | conserved hypothetical protein (168 aa) | 17248 | 29 | 0.2 | 3 |
| Afu5g12560 | Afu5g12560 | hypothetical protein (733 aa) | 83681 | 26 | 0.04 | 2 |
| Afu1g15080 | Afu1g15080 | hypothetical protein (620 aa) | 69179 | 26 | 0.05 | 1 |
| Afu7g06780 | Afu7g06780 | hypothetical protein (235 aa) | 26238 | 26 | 0.13 | 1 |
| Afu4g03730 | Afu4g03730 | dienelactone hydrolase (493 aa) | 54642 | 25 | 0.06 | 3 |
| Afu5g00230 | Afu5g00230 | hypothetical protein (583 aa) | 64230 | 25 | 0.05 | 2 |
| Afu1g10610 | Afu1g10610 | hypothetical protein (175 aa) | 19842 | 24 | 0.17 | 2 |
| Afu6g12310 | Afu6g12310 | conserved hypothetical protein (300 aa) | 33701 | 24 | 0.1 | 1 |
| Afu3g12340 | Afu3g12340 | conserved hypothetical protein (693 aa) | 75736 | 23 | 0.04 | 3 |
| Afu1g06060 | Afu1g06060 | hypothetical protein (1503 aa) | 164339 | 23 | 0.02 | 2 |
| Afu2g04140 | Afu2g04140 | conserved hypothetical protein (1560 aa) | 171043 | 23 | 0.02 | 4 |
| Afu1g12200 | Afu1g12200 | conserved hypothetical protein (313 aa) | 35110 | 23 | 0.09 | 1 |
| Afu4g13050 | Afu4g13050 | hypothetical protein (485 aa) | 54219 | 21 | 0.06 | 2 |
| Afu4g11600 | Afu4g11600 | hypothetical protein (845 aa) | 94168 | 21 | 0.03 | 2 |
| Afu4g08030 | Afu4g08030 | conserved hypothetical protein (625 aa) | 69539 | 19 | 0.05 | 2 |
| Afu2g04920 | Afu2g04920 | conserved hypothetical protein (1467 aa) | 154207 | 18 | 0.02 | 1 |
| Afu2g05940 | Afu2g05940 | conserved hypothetical protein (370 aa) | 40602 | 18 | 0.08 | 1 |
| Afu6g07380 | Afu6g07380 | conserved hypothetical protein (634 aa) | 65757 | 18 | 0.05 | 1 |
| Afu2g10690 | Afu2g10690 | MFS phosphate transporter, putative (667 aa) | 75128 | 17 | 0.04 | 1 |
| Afu2g09270 | Afu2g09270 | hypothetical protein (409 aa) | 45569 | 16 | 0.07 | 1 |
| Afu2g14260 | Afu2g14260 | conserved hypothetical protein (960 aa) | 102846 | 16 | 0.03 | 1 |
